# Nucleoside Drugs Induce Cellular Differentiation by Caspase-Dependent Degradation of Stem Cell Factors

**DOI:** 10.1371/journal.pone.0010726

**Published:** 2010-05-19

**Authors:** Tanja Musch, Yuva Öz, Frank Lyko, Achim Breiling

**Affiliations:** Division of Epigenetics, DKFZ-ZMBH Alliance, German Cancer Research Center, Heidelberg, Germany; University of Hong Kong, Hong Kong

## Abstract

**Background:**

Stem cell characteristics are an important feature of human cancer cells and play a major role in the therapy resistance of tumours. Strategies to target cancer stem cells are thus of major importance for cancer therapy. Differentiation therapy by nucleoside drugs represents an attractive approach for the elimination of cancer stem cells. However, even if it is generally assumed that the activity of these drugs is mediated by their ability to modulate epigenetic pathways, their precise mode of action remains to be established. We therefore analysed the potential of three nucleoside analogues to induce differentiation of the embryonic cancer stem cell line NTERA 2 D1 and compared their effect to the natural ligand retinoic acid.

**Methodology/Principal Findings:**

All nucleoside analogues analyzed, but not retinoic acid, triggered proteolytic degradation of the Polycomb group protein EZH2. Two of them, 3-Deazaneplanocin A (DZNep) and 2′-deoxy-5-azacytidine (decitabine), also induced a decrease in global DNA methylation. Nevertheless, only decitabine and 1β-arabinofuranosylcytosine (cytarabine) effectively triggered neuronal differentiation of NT2 cells. We show that drug-induced differentiation, in contrast to retinoic acid induction, is caused by caspase activation, which mediates depletion of the stem cell factors NANOG and OCT4. Consistent with this observation, protein degradation and differentiation could be counteracted by co-treatment with caspase inhibitors or by depletion of CASPASE-3 and CASPASE-7 through dsRNA interference. In agreement with this, OCT4 was found to be a direct *in-vitro*-target of CASPASE-7.

**Conclusions/Significance:**

We show that drug-induced differentiation is not a consequence of pharmacologic epigenetic modulation, but is induced by the degradation of stem-cell-specific proteins by caspases. Our results thus uncover a novel pathway that induces differentiation of embryonic cancer stem cells and is triggered by the established anticancer drugs cytarabine and decitabine. These findings suggest new approaches for directly targeting the stem cell fraction of human tumours.

## Introduction

Cancer is widely considered a developmental disease, caused by the destabilisation of the balance of cellular proliferation and differentiation [1], [2]. Tumours thus originate from cells with stem cell characteristics that have acquired aberrant gene expression patterns, mostly due to mutations and epimutations. These aberrant gene expression patterns lead to a block in differentiation and trigger uncontrolled proliferation. Loss of differentiation is thus an important characteristic of tumour cells and represents a defining feature of human cancers. As a consequence, differentiation therapy has been developed into an important approach for the treatment of cancers, particularly leukaemias [3], [4].

Stem cell features are controlled by a small group of transcription factors, like NANOG and OCT4, that maintain the expression of stem cell genes and repress the induction of differentiation-specific genes by recruiting repressive complexes of the Polycomb Group (PcG) [5], [6]. Differentiation-associated genes in mouse ES cells are repressed by PcG proteins and are often also marked by DNA methylation [7]. These repressive marks are successively lost during differentiation. A prominent example is the loss of PcG repression at the *HOXA* gene cluster during neuronal differentiation [8], [9]. It has also been shown that promoters of lineage-specific genes become methylated during differentiation, suggesting context-dependent interactions between DNA methylation and Polycomb repression [10]. A key component of PcG repression is the histone methyltransferase EZH2 (enhancer of zeste homolog 2), the enzymatic core component of the Polycomb repressive complex 2 (PRC2). This protein creates specific trimethylation patterns of lysine 27 of histone H3 (H3K27me3), which leads to concomitant transcriptional silencing [11], [12].

The characterization of drugs that modulate epigenetic processes and induce differentiation in human cancer cells represents an important aspect in the development of epigenetic cancer therapies. Retinoic acid (RA), which induces differentiation in many stem cell populations, was among the first substances used for differentiation therapy [3]. The seminal finding that the differentiation-inducing cytosine analogue 2′-deoxy-5-azacytidine (decitabine, DAC) acts as an effective inhibitor of DNA methyltransferases provided an important link between cellular differentiation and epigenetic regulation [13]. Another example is 3-Deazaneplanocin A (DZNep), which has been shown to cause proteolytic degradation of PRC2 components, to influence histone modification patterns and to induce moderate differentiation effects in acute myeloid leukaemia cells [14]–[17]. DZNep was originally synthesised as an inhibitor of S-adenosylhomocysteine hydrolase (SAHH), a key enzyme in S-adenosylmethionine (SAM) dependent methylation processes [18]. Treatment of MCF-7 breast cancer cells with DZNep led to the derepression of a defined set of Polycomb targets, which again suggested that the compound might also induce cellular differentiation [14]. While these findings proposed a close connection between epigenetic modulation and drug-induced differentiation, cytarabine (1 β-arabinofuranosylcytosine, araC), a cytosine analogue closely related to decitabine, effectively induces differentiation, without inhibiting DNA methylation [19]. Both decitabine and cytarabine have been shown to be effective in the treatment of myeloid leukaemias, a group of diseases that is characterised by a differentiation block of precursor cells [20]. However, it is still not clear how these substances induce cellular differentiation and whether the inhibition of epigenetic modifiers plays a significant role in these mechanisms.

Over the past few years, the human embryonic teratocarcinoma cell line NTERA2 D1 (NT2) has been established as an intriguing human cancer stem cell model and represents a valuable tool for the analysis of the mechanisms regulating cellular differentiation. We have used NT2 cells, which can be induced to differentiate with natural ligands, like retinoic acid [21], to characterise the differentiation-inducing mechanisms triggered by RA, araC, DAC and DZNep. The three nucleoside analogues caused degradation of EZH2, but drug-induced differentiation could be observed only for araC and DAC. The latter drugs became integrated into DNA and induced DNA damage, which triggered the caspase-dependent degradation of NANOG and OCT4. Our results suggest that drug-induced differentiation is not a consequence of pharmacologic inhibition of DNA methylation and/or histone methylation but caused by the direct degradation of stem cell factors. Our study uncovers a novel mechanism mediating cellular differentiation by nucleoside drugs and suggests that cytarabine and decitabine might be useful for targeting cancer stem cells.

## Results

### DAC and araC induce differentiation of NT2 EC cells

Embryonal carcinoma (EC) cells derived from teratocarcinomas show many features of pluripotent cells and are often considered to be the malignant counterpart of human embryonic stem cells [22]. The EC cell line NTERA D1 (NT2) is a subclone of the line TERA2, originally derived from a lung metastasis associated with a testicular germ cell tumour that was established from a nude mouse xenocraft [23]. NT2 cells have not only been shown to differentiate along the neuronal linage, but also have mesodermal and ectodermal lineage potential. NT2 cells thus represent a valuable stem cell and cancer stem cell model [22], [24]. Morphological neuronal differentiation of NT2 cells with RA usually requires several weeks of treatment [21]. Nevertheless, gene expression patterns change rapidly, and strong derepression of the anterior part of the PcG-repressed *HOXA* cluster becomes detectable after a few hours and is accompanied by significant epigenetic changes [8], [9].

**Figure 1:**
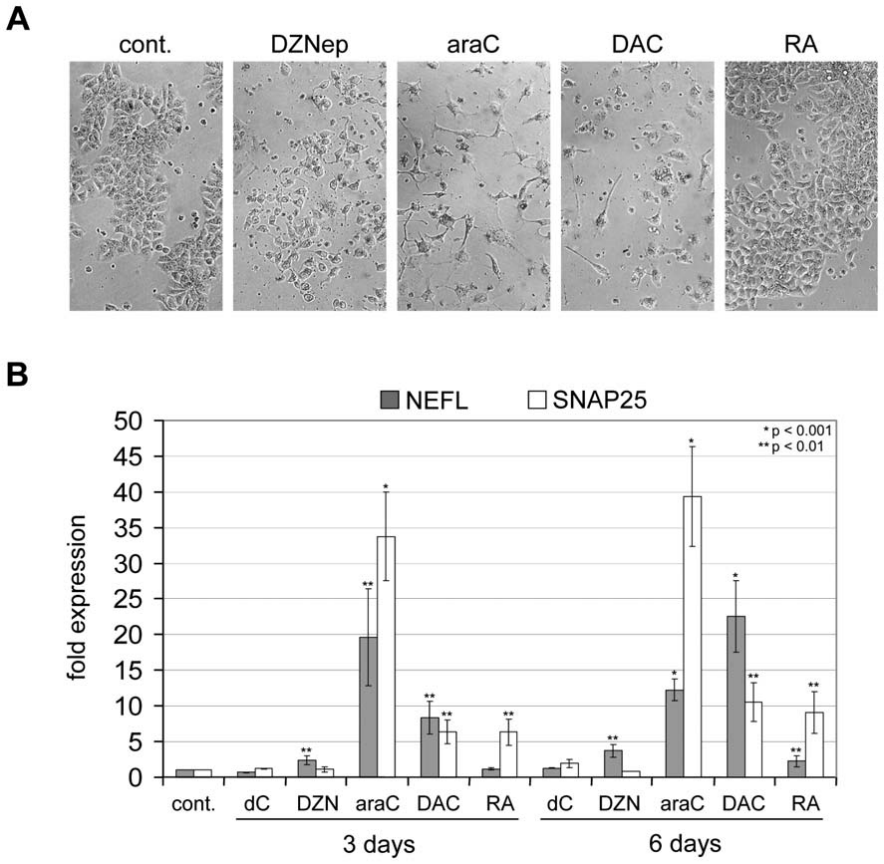
Nucleoside drugs araC and DAC induce neuronal differentiation of NT2 EC cells. (**A**) Microscopic images (10× magnification) of NT2 control cells (cont.) and NT2 cells treated with DZNep, araC, DAC or RA for 3 days. Neuron-like morphology appears most pronounced for araC and DAC. (**B**) qRT-PCR expression analysis of neuronal differentiation markers *NEFL* and *SNAP25* in drug-treated, RA- treated and control cells after 3 and 6 days of treatment. *NEFL* and *SNAP25* are prominently induced by araC and DAC. All qRT-PCR measurements were repeated at least three times and internally normalised to the corresponding *lamin-b* and *β-actin* expression levels. Deoxycytidine (dC) was included as a mock treatment control. Y-axis shows fold expression changes compared to the untreated control (cont.). P-values (Student's t-test) for the expression differences between control and treated cells for highly significant cases (p≤0.01) are shown. Asterisks indicate expression values that are significantly different from controls.

We assumed that activation of differentiation in NT2 cells would require substantial epigenetic modulation, especially changes in PcG presence, histone modification patterns and also DNA methylation. We thus chose DZNep, a drug that has been shown to cause degradation of the PcG protein EZH2 [14], and the established DNA methylation inhibitor DAC as potential drugs to induce differentiation. In addition, we used araC, an inhibitor of mitosis and potent inducer of apoptosis [25], as a structurally related drug with no direct epigenetic activity, and retinoic acid as a natural differentiation-inducing agent. All three nucleoside drugs showed similar, concentration-dependent cytotoxicity on NT2 cells and on other human cancer cell lines (Figure S1). For further treatments of NT2 cells we thus used drug concentrations equitoxic to the DZNep concentration established previously [5 µM, ref. 14], i.e. 20 µM for araC and 1 µM for DAC. Interestingly, we observed a neuron-like morphology in NT2 cells following incubation with araC and DAC already after 3 days, whereas treatment with DZNep and RA only led to weak or no morphological differentiation effects (Figure 1A). RT-PCR analysis of two neuronal markers known to be prominently induced in differentiating NT2 cells [26]–[28], *NEFL* (neurofilament light polypeptide) and *SNAP25* (synaptosomal-associated protein 25), demonstrated robust induction of both genes following treatment with araC or DAC (Figure 1B). DZNep and RA caused only minor activation of these genes, which is consistent with the absence of neuronal morphology after 3 days of treatment with both substances. These results establish araC and DAC, but not DZNep, as strong inducers of neuronal differentiation in NT2 cells.

### Analysis of drug-dependent changes in PcG repression

**Figure 2:**
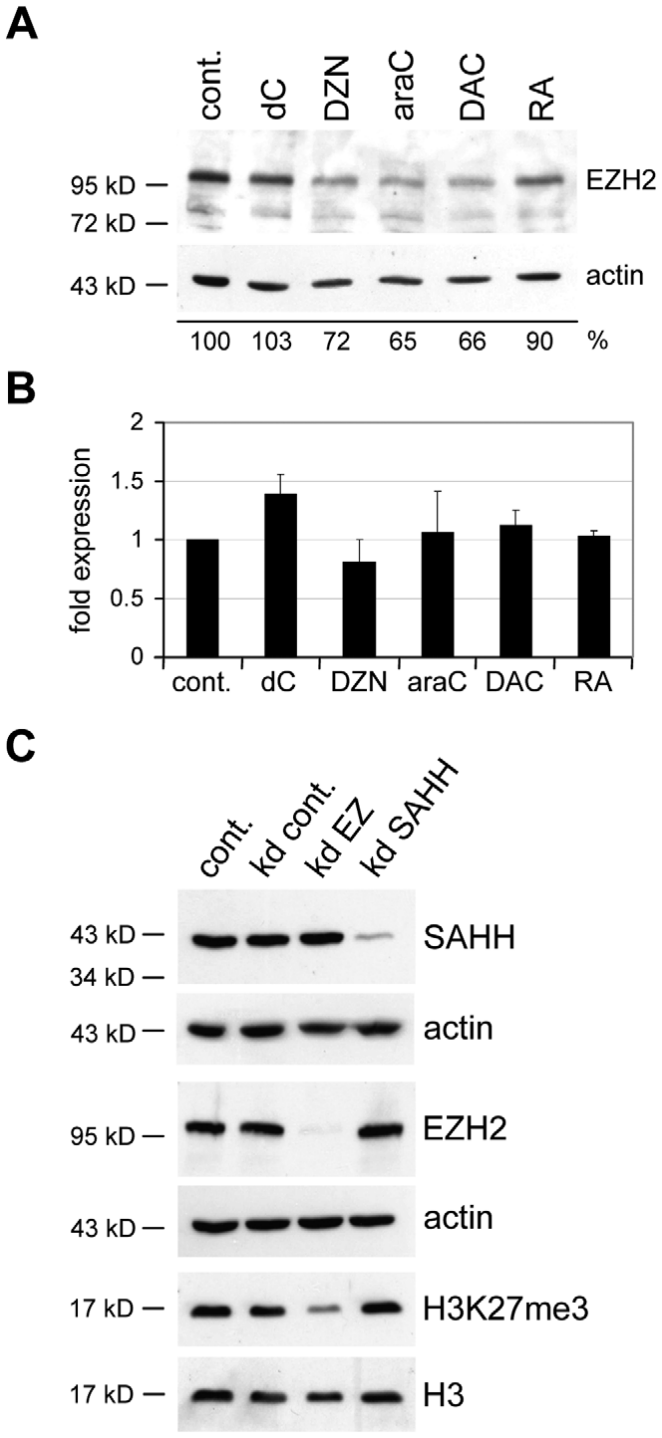
Nucleoside drugs affect EZH2 stability in NT2 cells. (**A**) Western blot showing EZH2 in control (cont.) cells and cells treated with dC, DZNep (DZN), araC, DAC and RA for 3 days. Reduced EZH2-levels were observed for araC, DAC and DZNep. β-actin was used as loading control, deoxycytidine (dC) as a mock treatment control. Below the blots intensities of the EZH2-bands, normalised to the corresponding β-actin bands, are indicated as percentage of the control (cont.). (**B**) *EZH2* mRNA expression levels in NT2 cells upon 3 days of drug treatment do not change significantly, as determined by quantitative RT-PCR. qRT-PCR measurements with at least three biological replicates were internally normalised to the corresponding *lamin-b* and *β-actin* expression levels. Deoxycytidine (dC) was included as a mock treatment control. (**C**) DZNep-mediated EZH2 depletion is not caused by SAHH inhibition. Western blot showing the effect of SAHH depletion (kd SAHH) on EZH2 and H3K27me3 levels in NT2 cells. Cells were treated for 72 hours with siRNAs. Knockdown of EZH2 (kd EZH2) was used as positive control, β-actin and histone H3 (H3) were used as loading controls. A mix of scrambled siRNAs was used as negative control (kd cont.). Reduction of SAHH-levels has no effect on EZH2 stability or H3K27me3 levels.

In order to identify the mechanisms that lead to drug-induced differentiation, we first analysed the effects on PcG repression. PRC2 degradation has been demonstrated for DZNep-treated MCF7 breast cancer cells [14] and the release from PcG repression is a molecular hallmark of differentiation [8], [9]. All three nucleoside drugs caused degradation of EZH2 after three days of treatment at equitoxic concentrations whereas RA had only a weak effect (Figure 2A). EZH2 degradation by these compounds could also be observed in other human cancer cell lines, although to varying degrees (Figure S2A). Drug treatment did not significantly affect the level of *EZH2* mRNA expression (Figure 2B), which confirmed that drug-dependent EZH2 degradation is regulated at the protein level [14].

It has been suggested that DZNep-mediated EZH2 degradation in MCF-7 cells might be related to the drug's ability to inhibit S-adenosylhomocyteine hydrolase (SAHH), a key enzyme in S-adenosylmethionine dependent methylation processes [14]. In order to determine the requirement of SAHH activity for EZH2 stability, we used siRNAs to efficiently deplete SAHH in NT2 cells. Subsequent Western analysis did not indicate any changes in the EZH2 protein level and in H3K27 trimethylation (Figure 2C). Similar results were also obtained in MCF-7 cells (Figure S2B). These findings indicate that the pharmacologic activity of DZNep is not limited to the inhibition of SAHH and that the molecular mechanisms triggering DZNep-induced EZH2 degradation remain to be established.

**Figure 3:**
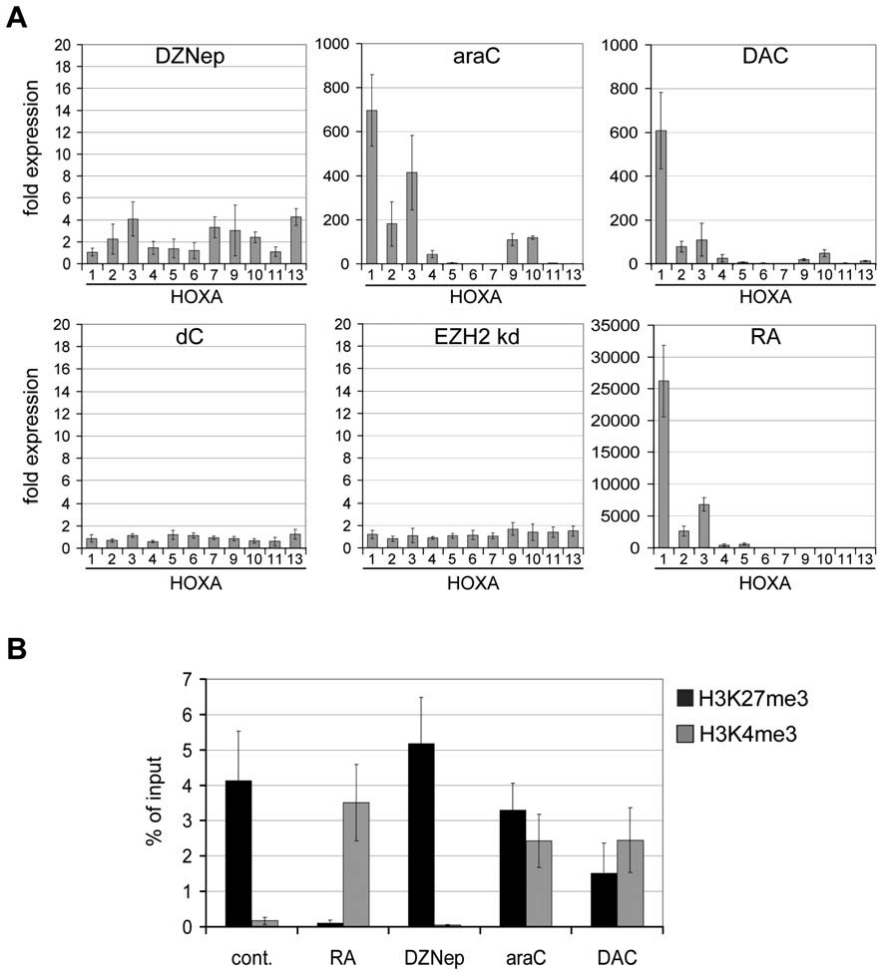
Nucleoside drugs affect Polycomb repression in NT2 cells. (**A**) qRT-PCR expression analysis of *HOXA* genes (1 to 13 - orthologs 8 and 12 are absent in the *HOXA*-cluster) after drug treatment for 3 days or knockdown of EZH2 (kd EZH2) for the same time period. Collinear activation of the cluster, similar to RA-treatment (positive control), could only be observed for araC- and DAC-treated NT2 cells. All treatments and measurements were repeated at least five times and internally normalised to the corresponding *lamin-b* and *β-actin* expression levels. Y-axis values indicate fold induction compared to the non-treated control. Knockdown of EZH2 was repeated three times in independent experiments (see Figure S3B). Deoxycytidine (dC) was included as a mock treatment control. (**B**) Chromatin immunoprecipitation (IP) analysis of the *HOXA1* promoter region in RA-, drug-treated and control NT2 cells. IP was repeated at least three times with chromatin from biological replicates using antisera specific for H3K27me3 and H3K4me3. Immunoprecipitated DNA was analyzed by real time PCR and a primer specific for the *HOXA1* promoter region. Enrichments are shown as percentage of the total input.

The 5′ genes of the *HOXA* cluster are among the first genes to be activated by RA and show rapid loss of PcG complexes and H3K27me3 upon in vitro differentiation [8], [9]. We therefore determined the expression levels of *HOXA* genes upon drug treatment. As expected, the natural ligand RA triggered strong expression in the 5′-region of the cluster (Figure 3A). DAC and araC also caused significant gene activation in a concentration-dependent manner, whereas DZNep-induced changes were relatively minor (Figure 3A and Figure S3A). These findings, together with the effects on EZH2 stability, suggested that RA, araC and DAC induce cellular differentiation independent of EZH2 degradation. This was confirmed by siRNA-mediated knockdown of EZH2, which only resulted in a very weak activation of the *HOXA* cluster (Figure 3A and S3B), indicating that EZH2 degradation is not sufficient for cellular differentiation.

Reactivation of *HOXA* genes by RA is accompanied by epigenetic changes in their histone modification patterns [8], [9]. Cytarabine and DAC have not been reported to affect global histone modifications. In contrast, DZNep has been shown to cause genome-wide loss of H3K27me3 [14] and additional repressive and active histone modifications [16]. We therefore analysed the effect of drug treatment on the histone methylation pattern of the *HOXA1* promoter by chromatin immunoprecipitation (ChIP). In control cells this region is marked by H3K27me3 and lacks the activating mark H3K4me3 (trimethylation of lysine 4 of histone H3), consistent with its silent state (Figure 3B). When *HOXA1* was induced by RA, the promoter was marked mainly by H3K4me3. DZNep did not cause any significant changes, whereas the *HOXA1*-activating drugs araC and DAC caused a substantial increase in H3K4me3, in parallel with a reduction in H3K27me3 levels (Figure 3B). This indicates that drug-induced activation of *HOXA1* is accompanied by histone modification changes, reflecting the loss of PcG repression at this target gene.

### Analysis of drug-dependent DNA methylation changes

**Figure 4:**
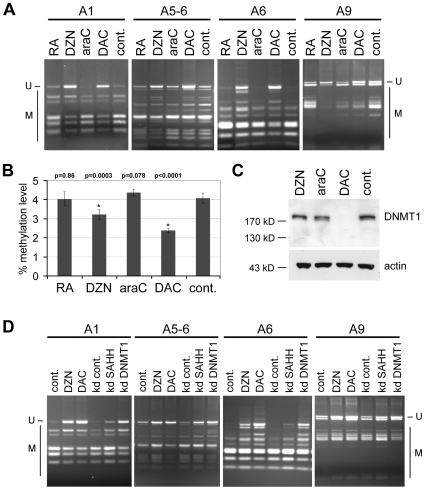
Nucleoside drugs cause differential DNA methylation changes in NT2 cells. (**A**) COBRA analysis of four CpG islands in the *HOXA* cluster in drug-treated and control NT2 cells, showing the demethylating activity of DAC and DZNep. “U” indicates unmethylated epialleles; “M” indicates methylated epialleles. (**B**) Genomic cytosine methylation levels decrease in DAC- and DZNep-treated NT2 cells. All measurements were repeated at least three times, standard deviations are indicated by error bars. P-values (Student's t-test) for the differences between control and treated cells are shown on top. Only treatment with DZNep (DZN) and DAC (columns marked with an asterisk) lead to highly significant changes (p≤0.01). (**C**) DNMT1 trapping assay in NT2 cells showing depletion of DNMT1 only after DAC-treatment. DNMT1 in drug-treated and control cells was detected by Western blot analysis of protein extracts from drug-treated cells. β-actin is shown as a loading control. (**D**) COBRA analysis of the same CpG islands as in (A) in DZNep- and DAC-treated cells, control cells and cells depleted for SAHH and DNMT1, showing that depletion of SAHH also leads to demethylation. “U” indicates unmethylated epialleles; “M” indicates methylated epialleles. Knockdown efficiencies for this experiment are shown in Figure S3C.

To further elucidate the pathways mediating drug-induced differentiation we also investigated DNA methylation. Previous studies have linked altered *HOX* gene expression patterns to changes in the DNA methylation status [29]–[31]. In addition, it has been suggested that DZNep might cause concomitant DNA methylation changes [14]. Lastly, DAC is an established inhibitor of DNA methylation and inhibition of DNA methylation has also been associated with cellular differentiation [13]. We therefore compared DZNep, araC, DAC and RA in their ability to alter *HOXA* methylation in NT2 cells. Methylation analysis by COBRA indicated dense DNA methylation of several *HOXA* CpG islands, some of which were demethylated upon incubation with DZNep or DAC, but not with araC or RA (Figure 4A). Similar results were also obtained using capillary electrophoresis to measure global cytosine methylation. Untreated NT2 cells showed a methylation level of 4.3%, which was not significantly altered after treatment with araC or RA (Figure 4B). However, treatment with DAC or DZNep significantly reduced global DNA methylation levels to 2.4% and 3.2%, respectively (Figure 4B). These results provide important confirmation for the DNA methylation inhibiting activity of DZNep [14], [16]. However, DZNep did not cause degradation of DNA methyltransferase 1 (DNMT1) (Figure 4C), which represents the main pathway for DAC-induced DNA demethylation [32]. Interestingly, depletion of SAHH by RNA interference (RNAi) also led to significant demethylation of *HOXA* CpG islands (Figure 4D and Figure S3C), which suggests that the demethylating activity of DZNep is a consequence of the inhibition of S-adenosylhomocysteine hydrolase. Our results thus confirm the demethylating activity of DZNep and suggest that DZNep-induced DNA demethylation is not related to DNMT1 degradation. Nevertheless, the lack of detectable araC-induced methylation changes suggests that inhibition of DNA methylation is not required for *HOXA* derepression and cellular differentiation.

### Drug-induced differentiation of NT2 cells requires degradation of stem cell factors

In our previous experiments we found differentiation by RA, araC and DAC to be accompanied by a loss of PcG-repression at the *HOXA*-cluster. However, RA did not trigger EZH2-depletion and EZH2-degradation by DZNep did not lead to *HOXA*-activation. This suggests that the drug-induced loss of PcG repression is caused by an upstream event. A recent study described the CASPASE-3-dependent degradation of the stem cell-specific transcription factor NANOG as important for RA-induced differentiation of human ES cells [33]. NANOG and the core transcription factor OCT4 maintain the expression of stem cell genes, and repress the induction of differentiation-specific factors [5]. A reduction of NANOG and OCT4 protein levels is thus a prerequisite for the induction of differentiation.

**Figure 5:**
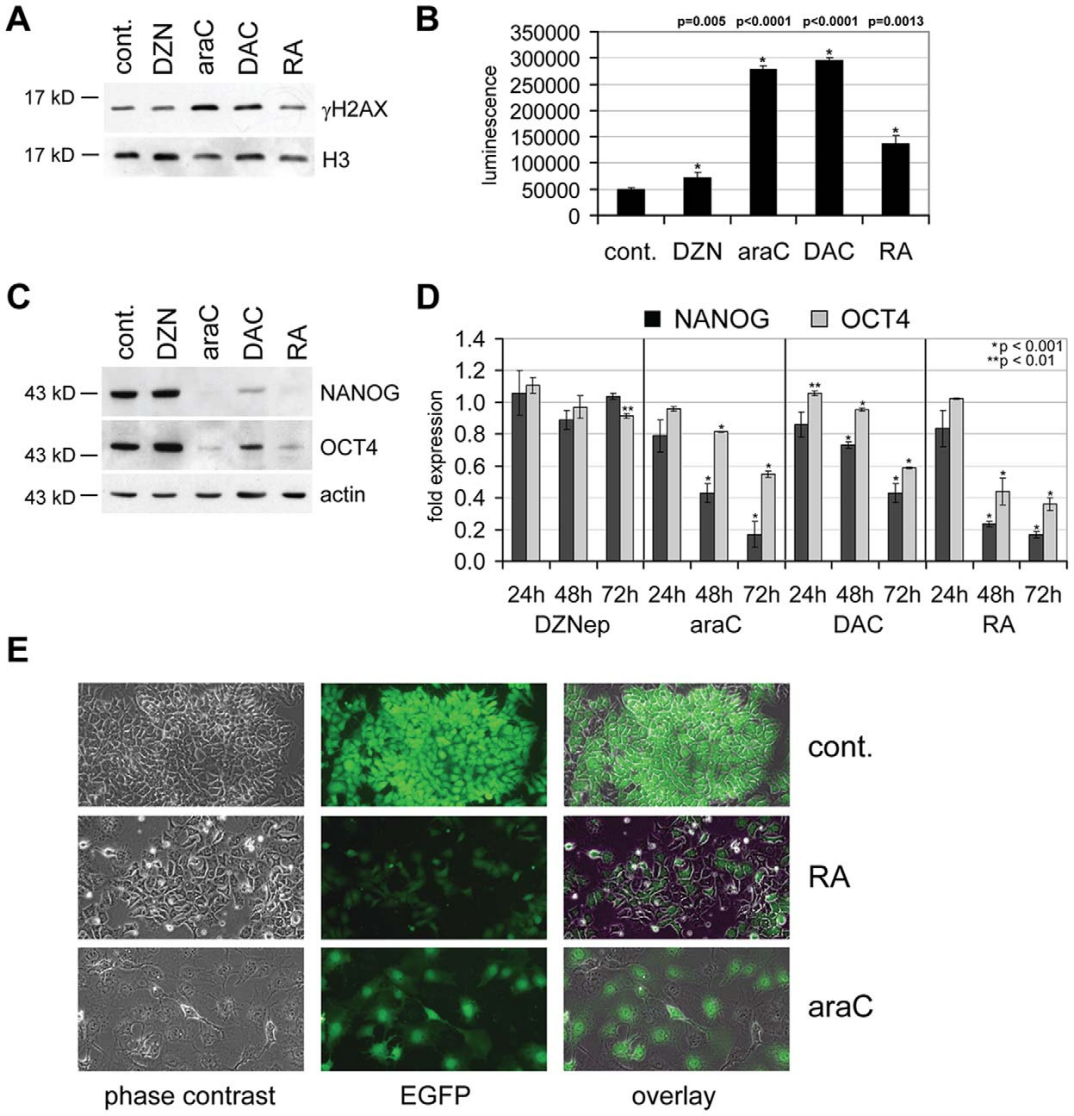
Differentiation-inducing drugs cause DNA damage in NT2 cells and deplete NANOG and OCT4 by caspase-dependent degradation. (**A**) Western blots showing the levels of γH2AX in untreated NT2 cells (cont.) and after 72h treatment with the indicated drugs. Histone H3 was used as loading control. (**B**) Caspase-Glo-assay of NT2 cells treated for 48 hours with DZNep (DZN), araC, DAC and RA. CASPASE-3/7-activity is shown as direct luminescence. Error bars represent standard deviations of at least 3 independent experiments. P-values (Student's t-test) for the differences between control and treated cells are shown on top. All observed changes are significant (marked with an asterisk). (**C**) Western blots showing the levels of NANOG and OCT4 in untreated NT2 cells (cont.) and after 72h treatment with the indicated drugs. β-actin was used as loading control. (**D**) Quantitative RT-PCR analysis of *NANOG* and *OCT4* after 24, 48 and 72 hours of treatment with DZNep, araC, DAC and RA. Diagrams show fold differences compared to the untreated control. All qRT-PCR measurements were repeated at least three times and internally normalised to the corresponding *lamin-b* and *β-actin* expression levels. P-values for the expression differences between control and treated cells for highly significant cases (p≤0.01) are shown. Asterisks indicate expression values that are significantly different from controls. (**E**) Microscopic images (10× magnification) of NT2 cells expressing EGFP under the control of the *OCT4*-promoter (first row) and the same cell line cells treated with RA (second row) and araC (third row) for 3 days. The first column shows the light microscopic images (phase contrast), the second row EGFP fluorescence, the third column an overlay. EGFP-signals persist in araC-treated cells.

Both araC and DAC, but not DZNep [14], are incorporated into DNA and have been shown to induce DNA damage, apoptosis and proteolytic cascades [25]. Consistently, we observed a robust accumulation of the DNA-damage marker γH2AX in cells treated with araC and DAC (Figure 5A). As DNA-damage is generally a trigger for caspase activation [34], [35], we next measured the enzymatic CASPASE-3/7 activity of drug-treated cells. Indeed, treatment with araC and DAC led to a substantial increase in caspase activity, while RA treatment had only a moderate effect and DZNep did not lead to clear caspase activation (Figure 5B). Of note, CASPASE-3/7 activation was paralleled by an effective depletion of NANOG and OCT4 (Figure 5C and S4A). DZNep had no effect on γH2AX, NANOG and OCT4, whereas RA-treatment did not lead to an increase of γH2AX, but also reduced both stem cell factors (Figure 5A, 5C and S4A). The lack of OCT4 reduction in DZNep-treated NT2 cells indicates that the mild morphological changes observed (Figure 1A) are not due to differentiation processes. Downregulation of *NANOG* and to lesser extent *OCT4* by araC and DAC was observed also at the mRNA level, while RA led to a very pronounced transcriptional repression of both genes (Figure 5D).

To further distinguish between protein degradation and regulation on the RNA level, we established a transfected NT2 cell line expressing EGFP under the control of the *OCT4* regulatory region [36]. Untreated cells strongly expressed EGFP, whereas treatment with retinoic acid for three days led to a significant reduction of the EGFP-signal (Figure 5E), indicating that *OCT4* expression is silenced on the mRNA level. Treatment with araC for the same period of time resulted in cellular differentiation, but the cells retained significant EGFP expression (Figure 5E), which is consistent with OCT4 downregulation at the protein level.

In order to test if direct depletion of OCT4 and NANOG induces differentiation of NT2 cells, we reduced the protein levels of OCT4, NANOG, EZH2, DNMT1 and SAHH by dsRNA interference and monitored neuronal morphology and expression of *SNAP25*. Depletion of SAHH, DNMT1, EZH2, NANOG did induce minor changes in *SNAP25* expression, but only the knockdown of OCT4 clearly induced neuronal differentiation, and, in parallel, significantly increased expression of *SNAP25* (Figure S5A and S5B). Combined knockdown of OCT4 and NANOG or OCT4, NANOG and EZH2, did not enhance the effect. *NANOG* expression is already strongly reduced in cells depleted for OCT4 (Figure S5B), as OCT4 is a major activator of the *NANOG* gene [5]. This also explains the lower levels of *NANOG* transcription upon drug treatment (Figure 5D). Nevertheless, depletion of stem cell factors, in particular OCT4, by RNAi shows similar effects on NT2 cells as treatment with araC and DAC and is sufficient to induce differentiation.

**Figure 6:**
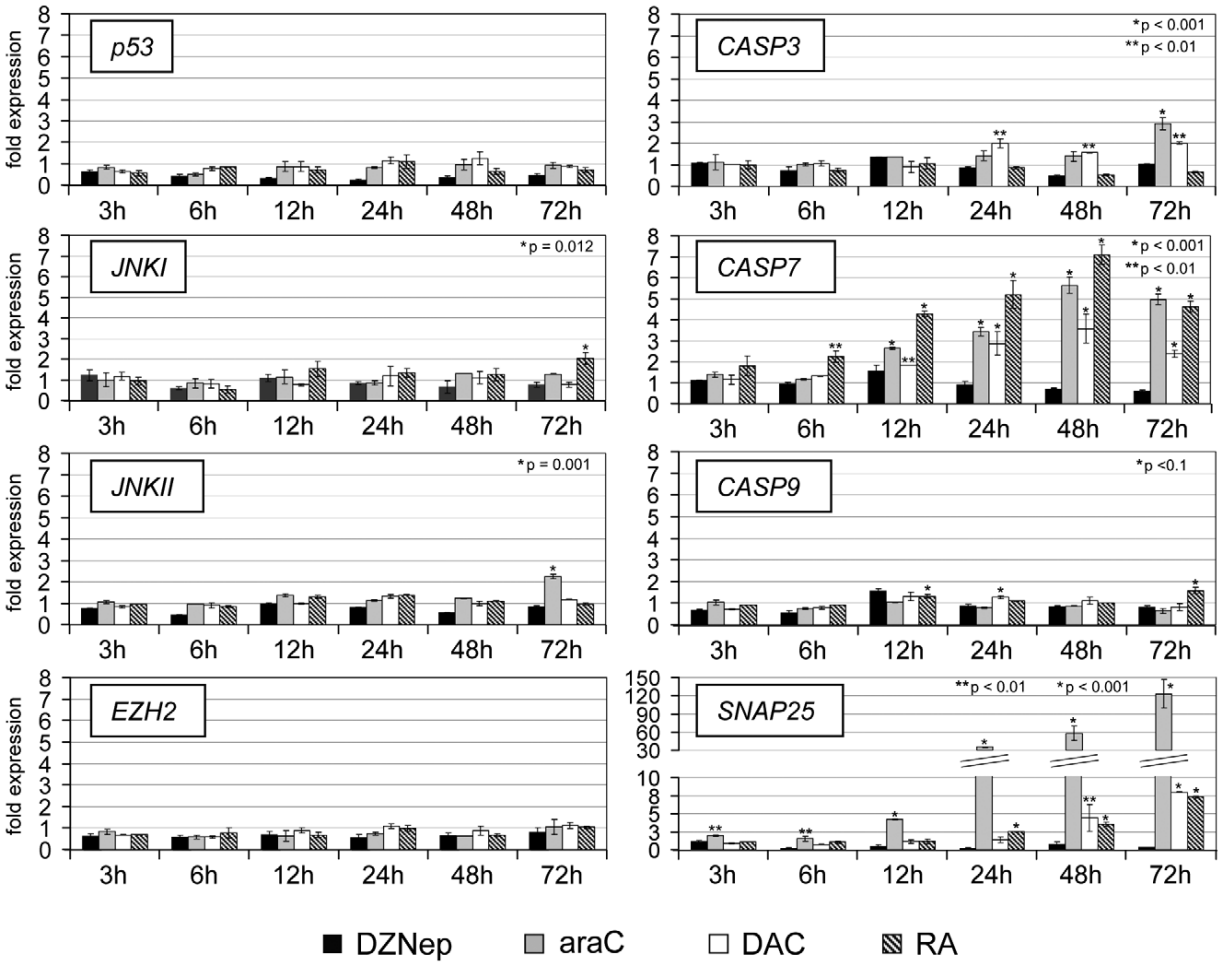
Expression analysis of key regulatory factors of proteolytic pathways. The graphs show a qRT-PCR expression analysis of *p53*, *CASPASE-3*, *CASPASE-7*, *JNKI*, *JNKII*, *EZH2* and the neuronal differentiation marker *SNAP25* in drug-treated NT2 cells. Expression values after 3, 6, 12, 24, 48 and 72 hours of treatment are shown as fold increase compared to untreated cells. All qRT-PCR measurements were repeated at least three times and internally normalised to the corresponding *lamin-b* and *β-actin* expression levels. P-values (Student's t-test) for the expression differences between control and treated cells for highly significant cases (p≤0.01) are shown. Asterisks indicate expression values that are significantly different from controls.

To confirm the involvement of proteolytic pathways in drug-induced NANOG and OCT4 degradation, we analysed the expression levels of several key regulatory factors of these pathways in a time course experiment by RT-PCR. As expected, we observed a significant induction of the *CASPASE-7* gene by araC and DAC, but also RA (Figure 6). Cytarabine and DAC also triggered moderate upregulation of *CASPASE-3* and the *c-Jun N-terminal kinase II* (*JNKII*). This was paralleled by the expression of the differentiation marker *SNAP25* (Figure 6). We observed no significant changes in gene expression for the apoptotic factor *p53*, for *c-Jun N-terminal kinase I* (*JNKI*), *CASPASE-9* and *EZH2* (Figure 6). Thus, CASPASE-7, and, to a lesser extent, CASPASE-3, seem to be critical factors in araC- and DAC-induced degradation processes.

### Drug-induced differentiation is caused by CASPASE-7-dependent degradation of OCT4

**Figure 7:**
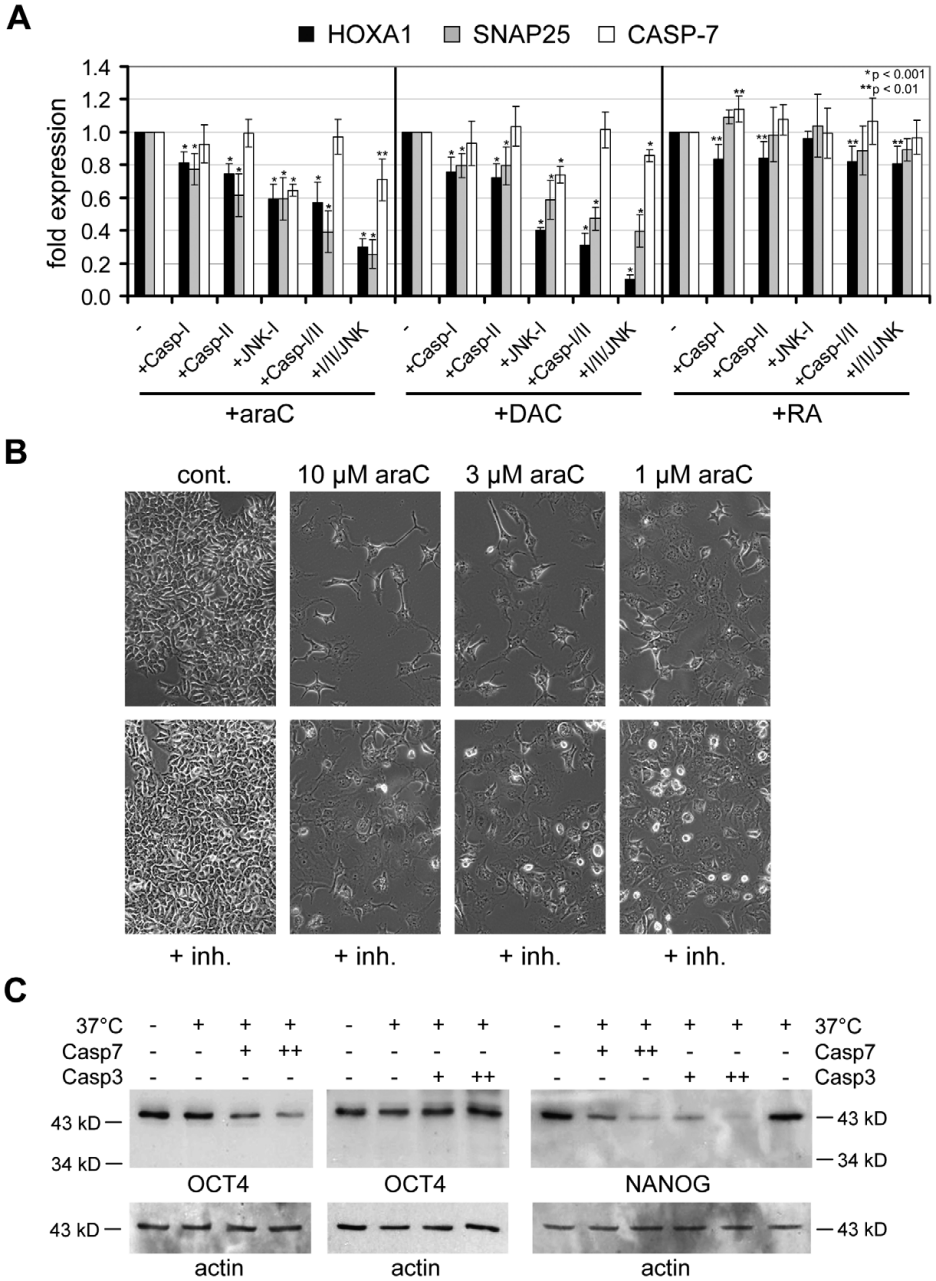
The effect of differentiation-inducing drugs in NT2 cells can be rescued by caspase and JNK inhibitors. OCT4 is a substrate of CASPASE-7. (**A**) Quantitative RT-PCR expression analysis of the differentiation markers *SNAP25*, *HOXA1* and of *CASPASE-7* after 3 days of treatment with araC, DAC or RA alone or in combination with caspase Inhibitors I and II (Casp-I, Casp-II), JNK inhibitor 1 (JNK), a combination of both caspase inhibitors (Casp-I/II) and a mixture of all three inhibitors (all). Diagrams show fold differences compared to araC-, DAC- and RA-treatment alone. Co-treatment with caspase inhibitors leads to reduced gene expression after araC and DAC treatment. All qRT-PCR measurements were repeated at least three times and internally normalised to the corresponding *lamin-b* and *β-actin* expression levels. P-values (Student's t-test) for the expression differences between only drug-treated (−) and co-treated cells for highly significant cases (p≤0.01) are shown. Asterisks indicate expression values that are significantly different from controls. (**B**) Microscopic images (10× magnification) of NT2 control cells (cont.) and NT2 cells treated for 72 h with 1, 3 and 10 µM araC (upper panels) and NT2 cells co-treated for 72 h with a mixture of all three inhibitors (lower panels) are shown. The araC-induced neuron-like morphology can be reverted to some degree by co-treatment with caspase and JNK inhibitors. (**C**) Western blots showing the levels of OCT4 and NANOG in nuclear extracts from NT2 cells without treatment (cont.), in nuclear extracts incubated at 37°C alone in the presence of 1 (+) or 2 (++) units of active recombinant human CASPASE-7 or CASPASE-3. β-actin was used as loading control. Antibodies specific for N-terminal peptides of OCT4 and NANOG were employed. OCT4 signals are efficiently reduced only by CASPASE-7, whereas NANOG is degraded by both caspases.

In order to further prove the role of caspases in drug-induced differentiation, we combined nucleoside drug treatment with caspase inhibitors (Inhibitor I and II) and a JNKII inhibitor (JNK Inhibitor I). These substances caused a significant reduction of the ability of araC or DAC to activate *HOXA1* and *SNAP25*, whereas RA-mediated induction of these genes and *CASPASE-7* expression was not significantly affected (Figure 7A). In addition, all three inhibitors partially rescued OCT4- and EZH2-degradation induced by araC (Figure S4B). No such effect could be observed for the reduction of OCT4 upon RA-treatment (Figure S4C), which is consistent with the notion that OCT4 is downregulated on the RNA level in RA-treated NT2 cells. DZNep has been shown to trigger proteasomal degradation of PRC2 components [14]. We thus also employed the proteasome inhibitor Lactacystin [37]. We observed no an effect on araC- or DAC-induced expression of *HOXA1* or *SNAP25* (Figure S6A) and Lactacystin did not rescue araC-induced OCT4 or EZH2 degradation (Figure S6B and S6C). In contrast, DZNep-induced EZH2 degradation was affected by Lactacystin (Figure S6C), which is in line with the established role of DZNep in inducing proteasome degradation [14].

The rescue effects exerted by caspase and JNK inhibitors on araC-induced differentiation could also be observed morphologically (Figure 7B). Combinatorial treatments with araC and caspase inhibitors led to clearly less differentiated cells compared to araC-treatment alone. Similar effects were observed when CASPASE-3 and CASPASE-7 were depleted by corresponding siRNAs in araC-treated cells (Figure S7A). NT2 cells induced to differentiate with araC showed significantly less differentiated cells when CASPASE-7 or both caspases were knocked down after 6 hours of araC-treatment and subsequent incubation for 48 hours (Figure S7A). In addition, combined knockdown of CASPASE-3 and -7 in araC-induced NT2 cells led to significantly lower activation of *HOXA1* and *SNAP25* (Figure S7B) and also partially restored OCT4 protein levels in araC-treated NT2 cells (Figure S7C).

In a final set of experiments we also tested if active recombinant human CASPASE-7 or CASPASE-3 is able to degrade OCT4 or NANOG in nuclear extracts of NT2 cells *in vitro*. As shown in Figure 7C, treatment with increasing amounts of CASPASE-7 led to a significant reduction of OCT4 signals, whereas similar treatment with CASPASE-3 had no effect on OCT4 stability (Figure 7C). In contrast, NANOG is degraded under similar conditions by both caspases, with CASPASE-3 showing a significantly stronger effect (Figure 7C). These experiments confirm that OCT4 and NANOG are direct substrates for CASPASE-7 and support published data showing that NANOG is targeted by CASPASE-3 [30].

Taken together, we identified CASPASE-7 and, to a lesser extent, CASPASE-3 as important mediators of araC- and DAC-induced OCT4 and NANOG degradation and drug-induced differentiation.

## Discussion

Differentiation-inducing compounds represent important tools for molecular biology and promising drugs for cancer therapy [4], [19], [38]. In this study, we compared the differentiation-inducing mechanism of three compounds - DZNep, cytarabine and decitabine - in a cancer stem cell model and identified a novel pathway for drug-induced differentiation. Importantly, our results show that drug-induced differentiation is not a consequence of pharmacologic inhibition of DNA methylation and/or histone methylation, but is triggered directly by the CASPASE-3/7-dependent degradation of stem cell specific proteins. In particular, we identified CASPASE-7 as a central enzyme specifically inducing OCT4 depletion. Cytarabine and decitabine have previously been reported to induce DNA damage and to trigger caspase- and JNK-dependent apoptotic pathways [25], [39]–[42]. Consistent with this notion, we can reverse the differentiation-associated effects caused by araC and DAC by using inhibitors of these pathways or by reducing levels of CASPASE-3 and CASPASE-7 by dsRNA interference.

The potential of cytarabine to induce differentiation has been known for a long time. The most prominent example is the use of the drug for the differentiation of bone marrow progenitor cells in leukaemias [4], [43]. Decitabine has first been described to induce differentiation more than 25 years ago in a landmark study linking DNA demethylation to cellular differentiation [13]. Both cytarabine and decitabine are incorporated into DNA and lead to double strand breaks (as monitored by the increase of hyperphosphorylated H2AX). Double strand breaks lead to the release of mitochondrial cytochrome c into the cytosol, which triggers apoptosome formation and mediates the activation of caspases-9, -3 and -7 [34], [35]. A recent publication describes that araC-induced differentiation of acute myeloid leukaemia cells positive for oncogenic RAS depends on p53 [44]. NT2 cells express p53 [45] and, even if we observed no effect of araC- or DAC-treatment on p53 expression, we can not rule out a role for p53 in drug-induced differentiation of NT2 cells. p53 is generally thought to trigger DNA repair and to halt the cell cycle upon DNA damage [34]. Nevertheless, p53 function can differ from cell type to cell type and accumulation of p53 can also activate pro-apoptotic genes, which finally leads to caspase activation [34].

We had initially speculated that the drug-induced degradation of EZH2 alone could lead to derepression of differentiation-specific genes in embryonic cancer stem cells. However, DZNep efficiently degraded EZH2 (without causing DNA damage in NT2 cells) and did not induce robust differentiation in our assays. The exact mechanism of DZNep-dependent degradation of EZH2 remains to be elucidated. It was assumed that DZNep-mediated inhibition of S-adenosylhomocysteine hydrolase (SAHH) leads to the proteasomal degradation of EZH2 [14]. However, we found that efficient knockdown of SAHH had no effect on EZH2 stability or H3K27 trimethylation. This indicates that the pharmacologic activity of DZNep is not limited to the inhibition of SAHH and that the molecular mechanisms triggering DZNep-induced EZH2 degradation remain to be established. Also, direct reduction of EZH2 by RNA interference did not trigger differentiation, induced only very weak *HOXA* expression in NT2 cells and also did not enhance the effects of OCT4 and NANOG depletion (Figure S5). It is possible that repressive PRC1 complexes remain attached to their target regions even in the absence of EZH2. Alternatively, reduced EZH2-repression could be compensated by its functionally redundant homologue EZH1 [46], [47]. Of note, our experiments confirm and extend earlier observations for a DNA demethylating activity of DZNep [14]. This activity could indeed be related to the DZNep-mediated inhibition of SAHH [18], as suggested by our finding that depletion of SAHH by RNA interference led to significant demethylation of *HOXA*-CpG-islands.

Degradation of OCT4 and NANOG (detectable already after 24 hours) clearly preceded EZH2 degradation, *HOXA* activation and upregulation of differentiation markers. Thus, degradation of PRC2 components is most likely a consequence of the depletion of stem cell markers, but nevertheless necessary for the subsequent differentiation-specific epigenetic changes and the progression of differentiation. As we show for retinoic acid, natural differentiation is slow, even if *OCT4* and *NANOG* are rapidly downregulated. This downregulation, however, appeared mainly to be caused by transcriptional repression. EGFP expression under the control of the *OCT4* regulatory region became clearly reduced after the addition of RA, whereas expression persisted in araC-treated cells, where transcriptional repression of the *OCT4* gene is much slower than proteolytic degradation of the gene product. In addition, RA-induced *OCT4* downregulation could not be rescued by caspase inhibitors. In line with these findings, OCT4-degradation was also not observed in RA-treated mouse embryonic stem cells [33]. We found that RA-induced *CASPASE-7*-expression was not paralleled by a similar increase in enzymatic activity, indicating that the enzyme remained mostly inactive, which is also consistent with the absence of EZH2 degradation in RA-treated cells at this time point. Significantly reduced EZH2-levels could only be observed after 2 weeks of RA-treatment (Figure S3D). Thus, PcG-dependent repression is only slowly removed in naturally differentiating NT2 cells, leading to a slow activation of neuronal markers like *NEFL* and *SNAP25*. In summary, it appears plausible that efficient and rapid differentiation, as induced by araC and DAC, requires the concomitant proteolytic degradation of stem cell factors and PcG proteins. Notably, the *NANOG* gene is activated by OCT4 [5] and reduced OCT4 will therefore also lead to reduced NANOG levels, as observed in our experiments.

In contrast to NANOG, OCT4 has so far not been described as a caspase substrate and is not cleaved by CASPASE-3 *in vitro* [33]. Our results indicate that CASPASE-7, but not CASPASE-3, can degrade OCT4 *in vitro*. This is in line with accumulating recent evidence showing that these two enzymes are functionally distinct [35], [48]. The CASVM web server for support-vector-machines-based prediction of caspase cleavage sites [49] predicts 5 cleavage sites in OCT4 (Figure S8). When overexposing the blots shown in Figure 7C, we observed a band of about 30 kD, which showed increased intensity in the lanes with the CASPASE-7-treated extracts (Figure S8C). As we observed this band only with an OCT4-antibody directed against an N-terminal peptide of the protein, but not with a C-terminal antibody (data not shown), it most likely represents a caspase-dependent N-terminal degradation product of OCT4. A proteolytic cut at the predicted P1-site 272 within the homeodomain of OCT4 would result in a N-terminal fragment of 30 kD (Figure S8B). This potential CASPASE-7 cleavage site needs to be confirmed in future experiments using recombinant OCT4 proteins bearing specific point mutations at this recognition sequence.

Stem cell characteristics are increasingly recognised as an important feature of human cancer cells and it is now widely assumed that a stem cell population plays a major role in the therapy resistance of tumours [50]–[52]. Strategies that target the stem cell properties of cancer cells will thus be critically important for translating this concept into therapeutical applications [3]. Our results uncover a novel pathway that triggers differentiation of embryonic cancer stem cells by the established anticancer drugs cytarabine and decitabine. These findings not only shed light on the mechanistic background of the biological effects caused by these drugs, but also show that cytarabine and decitabine might be useful in directly targeting the stem cell fraction of human tumours.

## Materials and Methods

### Cell culture and drug treatment

The human cell lines NT2 D1, MCF7, HeLa and A549 were maintained in Dulbecco's Modified Eagle Medium (D-MEM, with l-glutamine, 4500 mg/l d-glucose, without sodium pyruvat; Gibco) supplemented with 10% fetal bovine serum and 200 U/ml penicillin and 200 µg/ml streptomycin (all from Gibco) in a humidified atmosphere of 5% CO_2_ in air. Lymphoblast cell lines HL60 and HEL were kept in RPMI 1640 (Gibco), supplemented with 10% fetal bovine serum, 2mM l-glutamine and 200 U/ml penicillin and 200 µg/ml streptomycin (all from Gibco). NT2 cells were induced to differentiate with 10 µM all-trans retinoic acid (Sigma). Treatment of NT2 cells with araC, DAC, deoxycytidine (all Sigma) and DZNep (National Cancer Institute, NSC 617989) for 24, 48 or 72 hours was done under equitoxic conditions, i.e. 1 µM DAC, 20 µM araC, 20 µM dC and 5 µM DZNep (if not otherwise indicated). Substances, from aqueous stock solutions (5 or 50 mM), were added once to freshly seeded cells (2×10^5^ in a 6-well culture plate in 2 ml of medium). Treatment with JNK Inhibitor 1 (Alexis Biochemicals, 1 mM stock solution in water) at 1 µM, caspase Inhibitor I and II (Calbiochem, 5mM stock solution in DMSO) at 5 µM and with the proteasome inhibitor Lactacystin (Sigma L6785, 5 mg/ml stock solution in water) at 2 µM was done 2 hours before araC, DAC and RA were added. Inhibitors were then added every day. Cells were harvested by trypsin treatment and stored at −80°C.

### Viability assay

Cell viability was assayed using the CellTiter-Blue Cell Viability Assay (Promega). NT2, MCF7, HeLa or A549 cells were seeded into 96-well assay plates at a density of 1000 cells per well in 100 µl of medium. Test compounds araC, DAC, DZNep and dC were added in increasing concentrations from 40 nM up to 100 µM and cells were grown for 72 hours. Assays were performed in 6 biological replicates. After 3 days 20 µl of CellTiter-Blue reagent was added to each well, mixed and incubated for 1–4 hours. Absorbance at 570 and 600 nm was measured for each well and resazurin reduction was calculated according to the instructions of the kit. Viability was plotted as percentage compared to the untreated cells.

### RT PCR and real time analysis

Total RNA from drug-treated and untreated NT2 tissue culture cells was prepared using the Trizol-reagent (Invitrogen) according the manufacturer's specifications. Real-time RT-PCR was performed using the QuantiTect Reverse Transcription Kit (Qiagen). One µg of total RNA was processed in 20 µl reactions according to the manufacturer's specifications. One µl of the cDNA was used for a 10 µl PCR reaction using the Absolute QPCR SYBR Green Mix (Thermo Scientific) and a Roche LightCycler 480. PCR conditions: 1 cycle: 95°C×15 minutes; 42 cycles: 95°C×15 seconds, 60°C×40 seconds, read; melting curve 65°C–95°C, read every 1°C. Cycle threshold numbers for each amplification were measured with LightCycler 480 software and relative quantifications of expression status of the genes under observation comparing treated to non-treated cells were calculated and normalised using *lamin-b* and *β-actin* as internal standards. qRT-PCR measurements were repeated at least three times on biological replicates. P-values were calculated using Student's t-test. For primer sequences see Table S1.

### COBRA assay

For methylation analysis of CpG-rich regions in the *HOXA* cluster genomic DNA was treated with sodium bisulfite and analysed by COBRA [53]. PCR cycling conditions were 95°C for 30 seconds, 55–60°C for 30 seconds, and 72°C for 40 seconds for 35 cycles. The PCR products were gel purified and digested with BstUI or TaqIα (New England Biolabs, Beverly, MA). Digested PCR products were separated on 3% agarose gels. For primer sequences see Table S2.

### Capillary electrophoresis

Genomic DNA was prepared from cells using the DNeasy Kit (Qiagen). Global methylation levels were determined by capillary electrophoresis, as described previously [54]. Briefly, genomic DNA was enzymatically hydrolysed to single nucleotides and the nucleotides were derivatised with the fluorescent marker BODIPY (Molecular Probes). Derivatised nucleotides were separated by capillary electrophoresis and analysed in a Beckman PACE MDQ Molecular Characterization System. P-values were calculated using Student's t-test.

### DNMT1 trapping assay

NT2 D1 cells were grown and treated with the indicated concentrations of DZNep, araC and DAC as described above. After 3 days of treatment, cells were harvested, washed with PBS and lysed for 30 minutes on ice in trapping buffer (PBS pH 7.4, 0,1% SDS, 0,5% NP40, 0,5% Sodiumdeoxycholate, 1 mM DTT and Roche complete protease inhibitor cocktail). Insoluble debris was pelleted and supernatants were separated by 8% SDS-PAGE and transferred to polyvinylidene difluoride membranes (Millipore). DNMT1 was detected using an antibody from Santa Cruz Biotechnologies (C-17, sc-10222).

### Protein depletion via siRNAs

For depletion of SAHH, DNMT1, EZH2, NANOG and OCT4 ON-TARGETplus SMARTpool siRNAs (Dharmacon) were used. NT2 cells were seeded into 6-well plates at a density of 2×10^5^ in 2 ml of medium. siRNAs were transfected with the DharmaFECT1 transfection reagent (Dharmacon) according to the manufacturer's instructions. The final siRNA concentration was 50 nM. For combined knock downs the reactions were scaled up accordingly. After 48 h or 72 hours cells were retransfected (48 h), analysed by microscopy or harvested by trypsin treatment. Cell pellets were then analysed by Western blot and RT-PCR. Scrambled siRNAs for negative control experiments were also obtained from Dharmacon. For depletion of araC-induced CASPASE-3 and/or CASPASE-7 cells were treated for 6 hours with 10 µM araC and were then transfected with scrambled siRNAs, CASPASE-3 specific siRNAs, CASPASE-7 specific siRNAs and a mix of both as described above. After transfection the cells were grown for further 48 hours (without changing the medium), analysed by microscopy, then harvested by trypsin treatment and analysed by Western blot and RT-PCR.

### Western blotting and antibodies

Pellets of cultured cells were lysed in SDS-PAGE-buffer, shock frozen in liquid nitrogen and heated for 5 minutes at 95°C. Samples were separated on 8–10% SDS-PAGE and transferred to nitrocellulose (Whatman) or polyvinylidene difluoride (Millipore) membranes. Proteins were immunodetected using specific antibodies against EZH2 (Cell Signalling AC22), NANOG (abcam ab21624), OCT4 (abcam ab18976), β-actin (abcam ab8226) and SAHH (obtained from Michael Hershfield, Durham). Histones were separated using 15% SDS-PAGE gels and transferred to nitrocellulose (Whatman) membranes and immunodetected using specific antibodies against H3 (Cell Signaling 9715), H3K27me3 (Upstate/Millipore 07-449) and γH2AX (Milipore, clone JBW301). In some cases (Figures 2A and S2A) intensities of protein bands were background corrected and quantified using imageJ software (available at: http://rsb.info.nih.gov/ij/), normalised against the actin loading control and calculated as percentage of the signal for the untreated control.

### ChIP assay

Crosslinked chromatin was prepared and immunoprecipitated as described previously [9]. ChIP-grade antibodies specific for H3K27me3 (07-449) and H3K4me3 (07-473) were purchased from Upstate. Immunoprecipitates were finally dissolved in 30 µl of TE buffer (10 mM Tris-HCL pH 8, 1 mM EDTA). One µl was analysed by real time PCR using a primer pair specific for the *HOXA*1 promoter region (IPHOXA1prom_up ACTGGAAAGTTGTAATCCTATG; IPHOXA1prom_lo AGAAAGTTGGCACAGTCACG) in 10 µl PCR reactions, using the Absolute QPCR SYBR Green Mix (Themo Scientific) and a Roche LightCycler 480. PCR conditions: 1 cycle: 95°C×15 minutes; 42 cycles: 95°C×15 seconds/60°C×40 seconds, read; melting curve 65°C–95°C, read every 1°C. Cycle threshold numbers for each amplification were measured with LightCycler 480 software and enrichments were calculated as percentage of the input.

### Caspase assay

Activity of CASPASE-3 and -7 in DZNep-, araC-, DAC- and RA-treated NT2 cells was assayed with the Caspase-Glo 3/7 Assay System (Promega) according to the instructions of the manufacturer. P-values were calculated using Student's t-test.

### Preparation of nuclear extracts and caspase treatment

Nuclei from NT2 cells were prepared according to Wu [55]. The nuclear pellet was resuspended in lysis buffer (15 mM Tris-HCl pH 7.4, 150 mM KCl, 15 mM NaCl, 5 mM MgCl_2_, 0.1 mM EGTA, 1 mM DTT and Roche complete protease inhibitor cocktail; 1 ml per 10^6^ starting cells) and lysed by adding ammonium sulfate from a 4 M stock (pH 7,6) to a final concentration of 0.4 M for 15 minutes on ice. The lysate was centrifuged 30 minutes at 4°C and max. speed in a tabletop centrifuge and the supernatant was precipitated with solid ammonium sulfate (0.3 g per ml of supernatant). Proteins were pelleted at 4°C and max. speed in a tabletop centrifuge for 15 minutes. The protein pellet was dissolved in caspase buffer (50 mM Hepes pH 7,2, 50 mM NaCl, 0,1% CHAPS, 10 mM EDTA, 5% glycerol and 10 mM DTT), 10 µl per 10^6^ starting cells. Aliquots were stored at −80 °C or directly used for caspase digestion.

Aliquots corresponding to roughly 15 µg of total protein were treated for 20 minutes with 1 or 2 units of active recombinant human CASPASE-7 or CASPASE-3 (BioVision Research Products) in a total volume of 20 µl at 37°C. SDS-PAGE loading buffer (8×) was then added and the samples were separated on a 12% SDS-PAGE gel and transferred to nitrocellulose. Proteins were immunodetected using specific antibodies against NANOG (abcam ab21624), OCT4 (abcam ab18976, N-terminal peptide), OCT4 (abcam ab19857, C-terminal peptide) and β-actin (abcam ab8226).

### Histone preparation

Pellets of NT2-cells were washed once with cold PBS and then lysed on ice in cold lysis buffer (10 mM Tris pH 6.5, 5.2 g/l sodium bisulfite, 1% Triton-X100, 10 mM MgCl_2_, 486 g/l sucrose, 1,2 g/l sodium butyrate). Lysates were centrifuged for 5 minutes at 4°C, max. speed in a tabletop centrifuge. Pellets were washed 3× with cold lysis buffer and once with wash buffer (10 mM Tris-HCL pH 7.4, 13 mM EDTA). Final pellets were resuspended in 100 µl water, 1 µl H_2_SO_4_ was added and the samples were incubate on ice for 1 hour. Then, precipitates were pelleted and the supernatants were transferred to new tubes. Isolated histones were precipitated by adding 10 volumes of acetone at −20°C over night. Precipitates were collected and pellets were dissolved in water. After Bradford assay the histone solution was set to 1 µg/µl, stabilised by adding PAGE loading buffer to 1× and stored at −20°C.

### Generation of cell lines expressing OCT4-specific EGFP

Roughly 1×10^6^ NT2 cells were transfected with the plasmid phOCT4-EGFP, containing a 4 kb fragment of the human OCT4 regulative region (−3917 to +55) upstream of EGFP [35], using the Effectene Transfection Kit (Qiagen) according to the manufacturer's instructions. Cells were plated into 10 cm culture dishes and G148 (Sigma) selection was applied 24 hours after transfection at 350 µg/ml. After 2 weeks of selection surviving colonies were picked and expanded. One clone, showing approximately 90% of EGFP positive cells, was chosen for treatment with araC and RA.

## Supporting Information

Figure S1 Nucleoside drugs affect the viability of MCF7, NT2, HeLa and A549 cells in a similar manner. Cells were treated with increasing concentrations of DZNep, araC, DAC (40 nM up to 100 µM) and viability was analysed via resazurin reduction after 3 days. Error bars represent standard deviations; deoxycytidine (dC) was included as a mock treatment control.
(5.78 MB TIF)

Figure S2 Effects of drug-treatment on EZH2-stability. DZNep-mediated EZH2 depletion is not caused by SAHH inhibition. (A) Western blots showing drug-induced degradation of EZH2 in the cancer cell lines MCF7, A549, HeLa, HEL and HL60. Total extracts from cells treated with DZNep, araC and DAC for three days were analysed. β-actin was used as loading control. Below the blots intesities of the EZH2-bands, normalised to the corresponding β-actin bands, are indicated as percentage of the control (cont.). (B) Western blot showing the effect of SAHH depletion (kd SAHH) on EZH2 and H3K27me3 levels in MCF7 cells. Cells were treated for 72 hours or 2 times 48 hours with siRNAs. Knock down of EZH2 (kd EZH2) was used as positive control; β-actin was used as a loading control. A scrambled siRNA was used as negative control (kd cont.). Reduction of SAHH-levels has no effect on EZH2 stability or H3K27me3 methylation.
(5.42 MB TIF)

Figure S3 Effects of drug-treatment on HOX expression in NT2 cells. Efficiency of RNAi. Effect of RA-treatment on EZH2-stability. (A) qRT-PCR expression analysis of HOXA1, HOXA2 and HOXA3 in NT2 cells after 3 days of drug incubation. Drug-treatment at increasing concentrations (40 nM up to 20 µM) was done for 3 days and corresponding total RNA was reverse transcribed and analysed. Expression of all three HOX genes increases with drug concentration. Y-axis values indicate fold induction compared to the non-treated control. Deoxycytidine (dC) was included as a mock treatment control. All qRT-PCR measurements were repeated at least three times and internally normalized to the corresponding lamin-b and β-actin expression levels. Error bars represent standard deviations. (B) EZH2 transcription is efficiently reduced after siRNA mediated knock down in NT2 cells. EZH2 expression was quantified by qRT-PCR analysis in three independent knockdown experiments. Y-axis values indicate fold reduction compared to the scrambled control (kd cont.). (C) SAHH and DNMT1 transcription is efficiently reduced after siRNA mediated knock down in NT2 cells. Expression was quantified by qRT-PCR analysis in the knockdown experiment used for the COBRA analysis shown in figure 4D. Y-axis values indicate fold reduction compared to the scrambled control (kd cont.). (D) Western blot showing the effect of RA-treatment on the stability of EZH2 in NT2 cells during one month of treatment. Reduced protein levels could be observed after 3 weeks. β-actin was used as loading control.
(5.08 MB TIF)

Figure S4 Differentiation-inducing drugs deplete NANOG and OCT4 by caspase-dependent degradation in NT2 cells. (A) Western blots showing levels of NANOG and OCT4 proteins in cells treated for 6, 12, 24 and 48 hours with araC, DAC and RA. β-actin was used as a loading control. The blot showing retinoic acid induced changes (on the right) was reprobed after NANOG-staining with OCT4 antibodies. Thus the β-actin control is the same for this experiment. DAC- and araC-treated OCT4 and NANOG blots are from two different experiments. (B) Western blots showing that drug-induced degradation of OCT4 and EZH2 in NT2 cells after 36 hours (OCT4) and 72 hours (EZH2) of treatment with araC is reduced (the signal is restored) by caspase or JNK inhibitors. β-actin served as loading control. (C) Western blots showing that RA-induced reduction of OCT4 48 hours of treatment with retinoic acid is not significantly reduced by caspase inhibitors I and II (Casp-I, Casp-II), JNK inhibitor 1 (JNK), a combination of both Caspase inhibitors (Casp-I/II) and a mixture of all three inhibitors (all). β-actin was used as loading control.
(5.11 MB TIF)

Figure S5 Depletion of stem cell factors induces differentiation of NT2 cells. (A) Microscopic images (10× magnification) of NT2 control cells (cont.) and NT2 transfected with a mix of scrambled siRNAs (kd cont.), and siRNAS specific for DNMT1 (D), SAHH (S), EZH2 (E), NANOG (N), OCT4 (O) and various combinations of these. After transfection the cells were grown for 72 hours. Knock down of OCT4 clearly induces neuronal phenotypes in NT2 cells. (B) Quantitative RT-PCR expression analysis of the differentiation marker SNAP25 and of DNMT1 (D), SAHH (S), EZH2 (E), NANOG (N) and OCT4 (O) after the knock down experiment described in (A). Diagrams show fold differences compared to control cells (cont.). The transcription of DNMT1, SAHH, EZH2, NANOG and OCT4 is efficiently reduced after the respective siRNA mediated knock down. Expression of the differentiation marker SNAP25 is weakly induced by NANOG knock down, but significantly upregulated upon depletion of OCT4, which is in line with the morphological observations. A combined knock down of NANOG and OCT4, or NANOG, OCT4 and EZH2 does not enhance the effect. qRT-PCR measurements are from two independent experiments and were internally normalised to the corresponding lamin-b and β-actin expression levels. P-values (Student's t-test) for the expression differences between untreated cells (cont.) and transfected cells for highly significant cases (p≤0.01) are indicated. Asterisks indicate expression values that are significantly different from controls.
(5.49 MB TIF)

Figure S6 The effects of araC and DAC can not be rescued by the proteasome inhibitor Lactacystin. (A) Quantitative RT-PCR expression analysis of the differentiation markers SNAP25 and HOXA1 in NT2 cells after 3 days of treatment with araC, DAC or RA alone or in combination with the proteasome inhibitor Lactacystin (LAC) or a combination of two caspase inhibitors (Casp-I/II). Diagrams show fold differences compared to araC-, DAC- and RA-treatment alone. Co-treatment with caspase inhibitors leads to reduced gene expression after araC- and DAC-treatment, whereas Lactacystin had no significant effect. All qRT-PCR measurements were repeated at least three times and internally normalised to the corresponding lamin-b and β-actin expression levels. P-values (Student's t-test) for the expression differences between only drug-treated and co-treated cells for highly significant cases (p≤0.01) are indicated. Asterisks indicate expression values that are significantly different from controls. (B) Western blots showing that drug-induced degradation of OCT4 in NT2 cells after 36 hours of treatment with araC is reduced (the signal is restored) by the mix of caspase inhibitors (Casp), but not by treatment with Lactacystin (LAC, middle). β-actin served as loading control. (C) Western blots showing that drug-induced degradation of EZH2 in NT2 cells after 72 hours of treatment with araC is reduced (the signal is restored) by the mix of caspase inhibitors (Casp), but not by treatment with Lactacystin (LAC, middle). In contrast, DZNep-induced degradation of EZH2 is also inhibited by Lactacystin (blot on the right). β-actin served as loading control.
(4.70 MB TIF)

Figure S7 The effect of differentiation-inducing drugs in NT2 cells can be rescued by depletion of CASPASE-3 and -7. (A) Microscopic images (10× magnification) of NT2 control cells (cont.) and NT2 cells treated for 54 h with 10 µM araC (araC). The remaining images show cells that were treated for 6 hours with 10 µM araC, then were transfected with a mix of scrambled siRNAs (kd cont.), CASPASE-3 specific siRNAs (kd CASP-3), CASPASE-7 specific siRNAs (kd CASP-7) and a mix of both (kd CASP-3/7). After transfection the cells were grown for further 48 hours (with araC present). The araC-induced neuron-like morphology can be reverted to some degree by the double knock down. (B) Quantitative RT-PCR expression analysis of the differentiation markers SNAP25, HOXA1 and of CASPASE-3 and CASPASE-7 after the treatment described in (A). Diagrams show fold differences compared to araC-treatment alone. CASPASE-3 and CASPASE-7 transcription is efficiently reduced after the respective siRNA mediated knock down. Combined knock down of both caspases leads to a significantly reduced araC-induced expression of SNAP25, HOXA1. qRT-PCR measurements are from two independent experiments and were internally normalised to the corresponding lamin-b and β-actin expression levels. P-values (Student's t-test) for the expression differences between only araC-treated (cont.) and transfected cells for highly significant cases (p≤0.01) are indicated. Asterisks indicate expression values that are significantly different from controls. (C) Western blots showing that araC-induced degradation of OCT4 (under the conditions described in A) is reduced (the signal is restored) by combined depletion of CASPASE-3 and CASPASE-7. β-actin served as loading control.
(4.08 MB TIF)

Figure S8 Potential caspase sites in the OCT4 protein. (A) Predicted caspase substrate cleavage sites by the CASVM (server for SVM Prediction of Caspase Substrates Cleavage Sites - http://casbase.org/casvm/index.html) using the P4-P2′and P14-P10′-trained classifiers are listed. The four amino acid core sequence (P4-P1) and the position of the P1-site along the 360 amino acid sequence of OCT4 are shown for each potential or predicted site. (B) The positions of the 5 predicted cutting sites are indicated below a sketch of the OCT4 protein. The POU-domain and the homeodomain are highlighted. The calculated molecular weight in kD for potentially originating N-terminal fragments after caspase digest are indicated below the arrows. The potential site used by CASPASE-7 is marked with an asterisk (C) Western blots showing the levels of OCT4 in nuclear extract from NT2 cells without treatment (cont.), in nuclear extract incubated at 37°C or treated with 1 (+) or 2 (++) units of active recombinant human Caspase-7 at 37°C. An antibody specific for an N-terminal peptide of OCT4 was used. β-actin was used as loading control. The blot is identical to the one shown in Fig. 7C but was exposed longer. A digestion product of ca. 30 kD becomes visible, that is significantly increased upon CASPASE-7 digestion (arrow with asterisk). This would correlate with a cut at the predicted site within the homeodomain of OCT4 (asterisk in B).
(5.86 MB TIF)

Table S1 RT-primer pairs used in this study.
(0.04 MB DOC)

Table S2 COBRA primer pairs used in this study.
(0.03 MB DOC)
