# Supplementary figures and images for: Nucleoside Drugs Induce Cellular Differentiation by Caspase-Dependent Degradation of Stem Cell Factors
Source: PLoS One. 2010 May 19;5(5):e10726. doi: 10.1371/journal.pone.0010726 (PMC2873290; doi:10.1371/journal.pone.0010726)

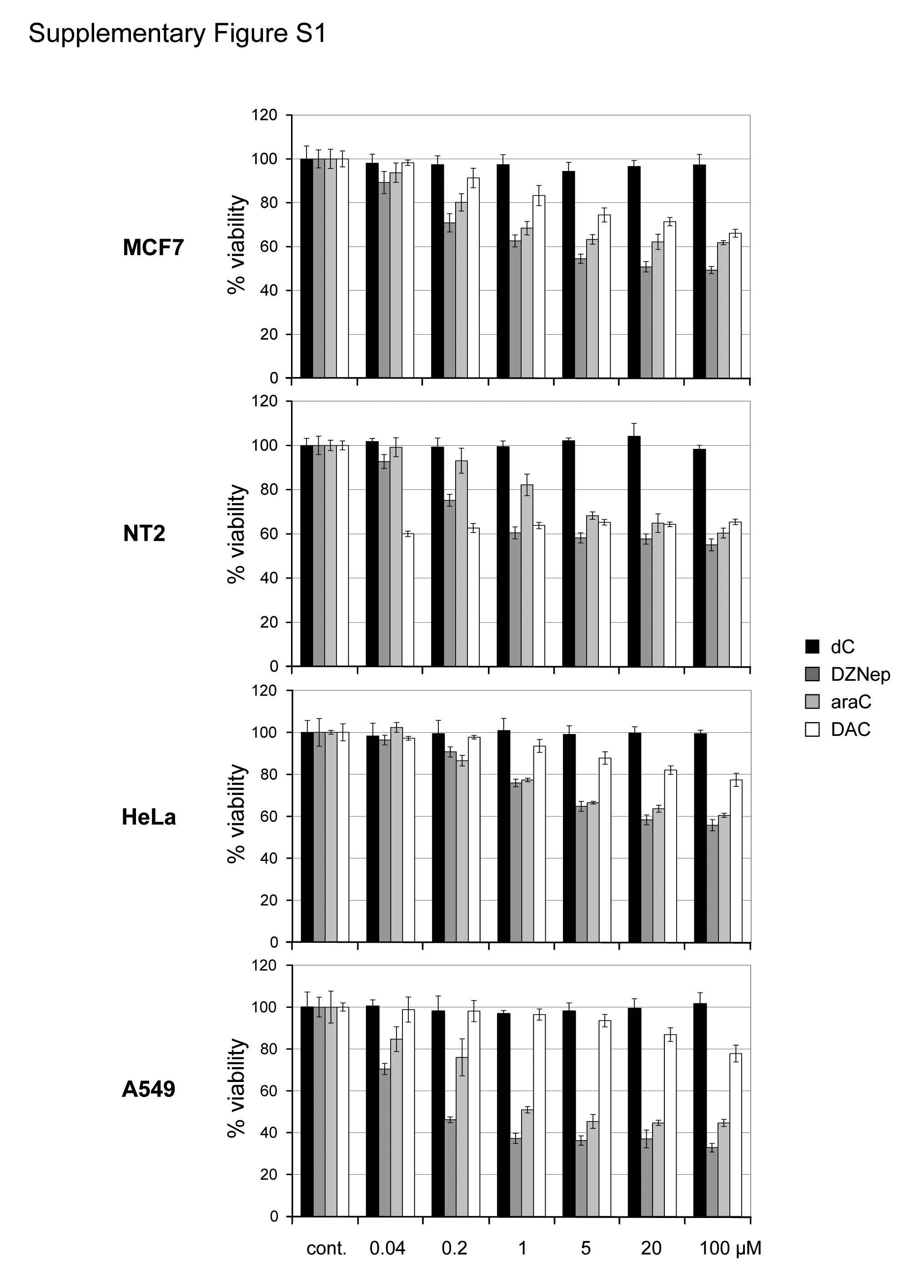

Supplement: Figure S1 — Nucleoside drugs affect the viability of MCF7, NT2, HeLa and A549 cells in a similar manner. Cells were treated with increasing concentrations of DZNep, araC, DAC (40 nM up to 100 µM) and viability was analysed via resazurin reduction after 3 days. Error bars represent standard deviations; deoxycytidine (dC) was included as a mock treatment control. (5.78 MB TIF) [file pone.0010726.s001.tif]

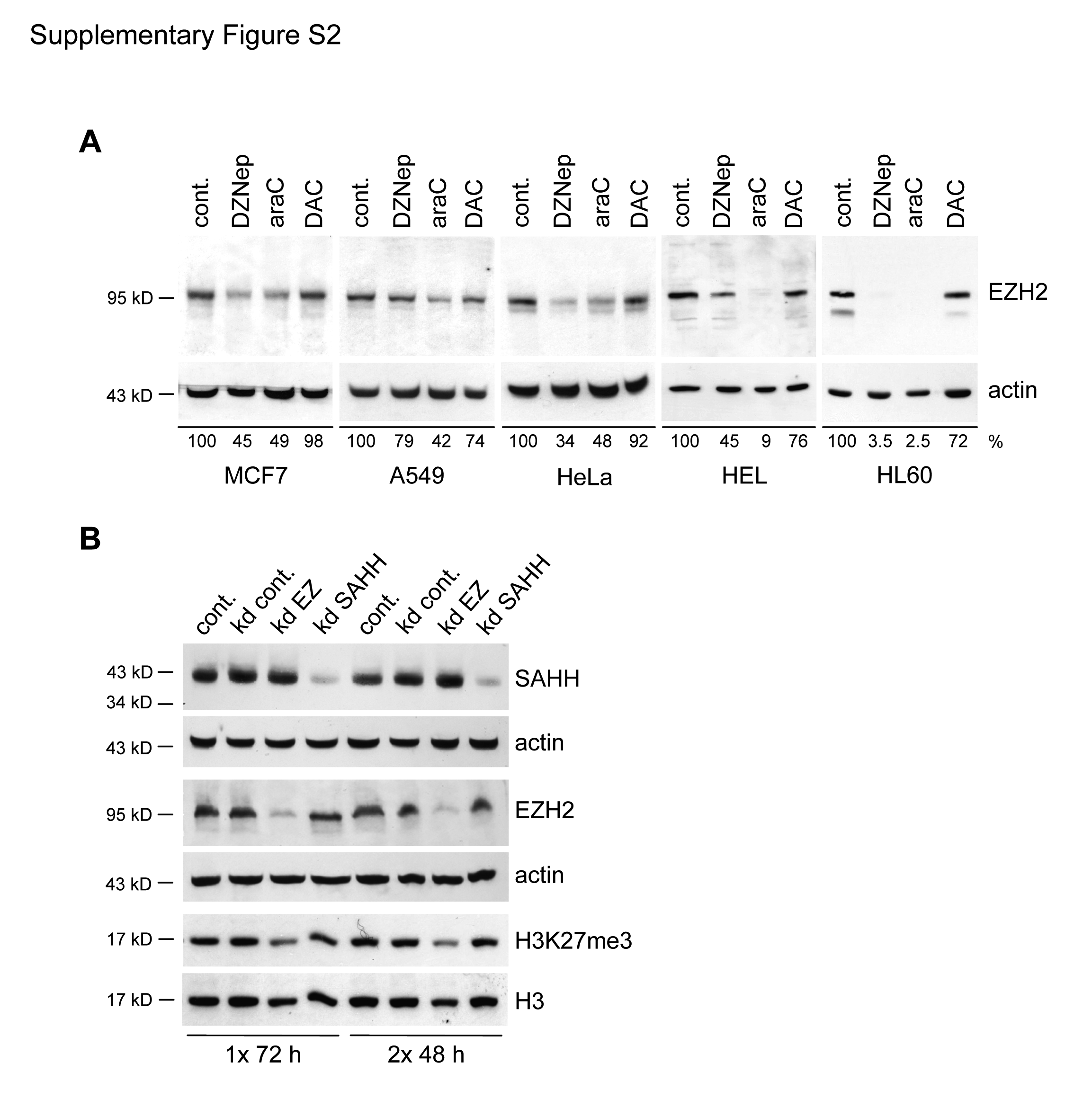

Supplement: Figure S2 — Effects of drug-treatment on EZH2-stability. DZNep-mediated EZH2 depletion is not caused by SAHH inhibition. (A) Western blots showing drug-induced degradation of EZH2 in the cancer cell lines MCF7, A549, HeLa, HEL and HL60. Total extracts from cells treated with DZNep, araC and DAC for three days were analysed. β-actin was used as loading control. Below the blots intesities of the EZH2-bands, normalised to the corresponding β-actin bands, are indicated as percentage of the control (cont.). (B) Western blot showing the effect of SAHH depletion (kd SAHH) on EZH2 and H3K27me3 levels in MCF7 cells. Cells were treated for 72 hours or 2 times 48 hours with siRNAs. Knock down of EZH2 (kd EZH2) was used as positive control; β-actin was used as a loading control. A scrambled siRNA was used as negative control (kd cont.). Reduction of SAHH-levels has no effect on EZH2 stability or H3K27me3 methylation. (5.42 MB TIF) [file pone.0010726.s002.tif]

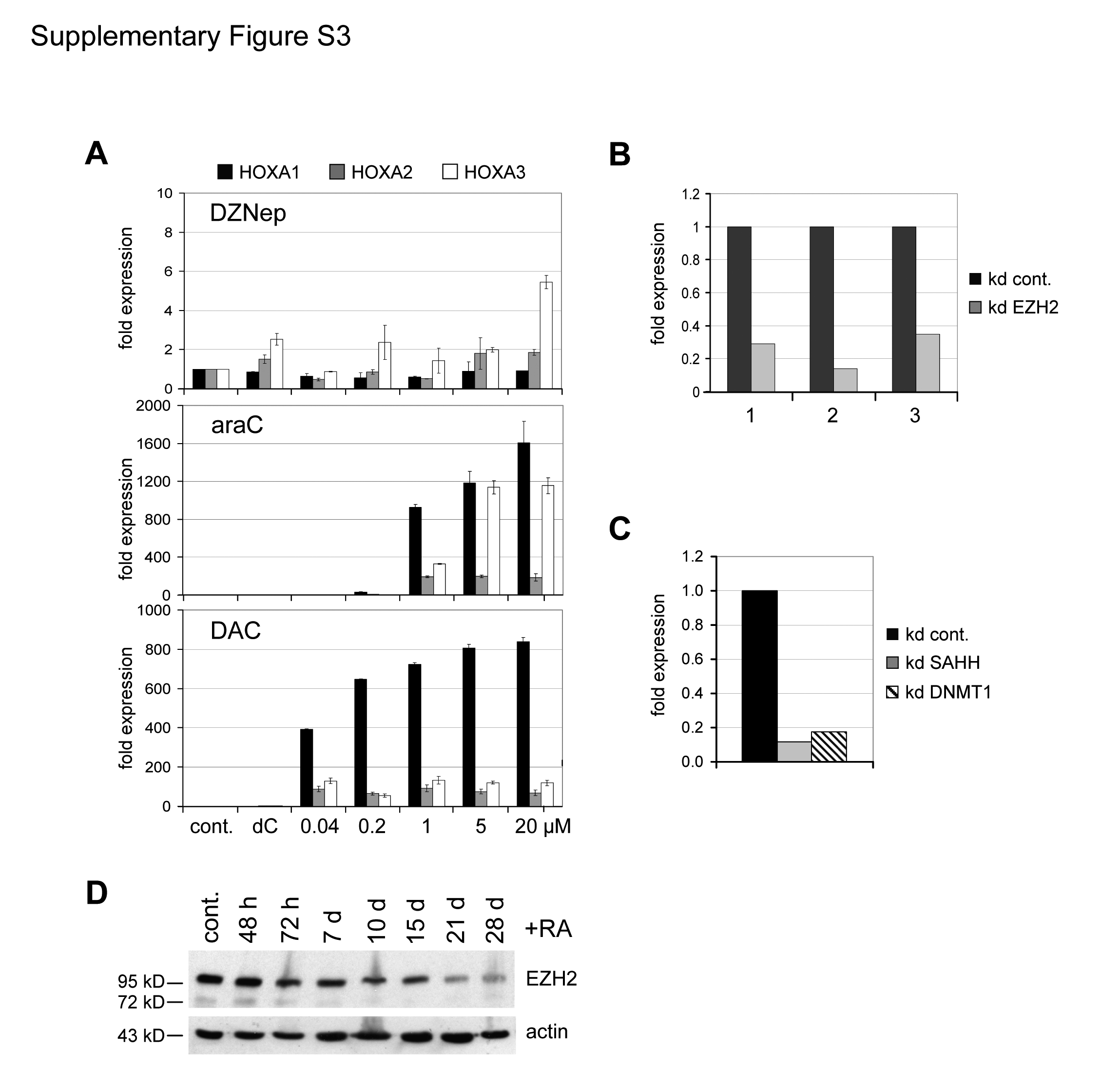

Supplement: Figure S3 — Effects of drug-treatment on HOX expression in NT2 cells. Efficiency of RNAi. Effect of RA-treatment on EZH2-stability. (A) qRT-PCR expression analysis of HOXA1, HOXA2 and HOXA3 in NT2 cells after 3 days of drug incubation. Drug-treatment at increasing concentrations (40 nM up to 20 µM) was done for 3 days and corresponding total RNA was reverse transcribed and analysed. Expression of all three HOX genes increases with drug concentration. Y-axis values indicate fold induction compared to the non-treated control. Deoxycytidine (dC) was included as a mock treatment control. All qRT-PCR measurements were repeated at least three times and internally normalized to the corresponding lamin-b and β-actin expression levels. Error bars represent standard deviations. (B) EZH2 transcription is efficiently reduced after siRNA mediated knock down in NT2 cells. EZH2 expression was quantified by qRT-PCR analysis in three independent knockdown experiments. Y-axis values indicate fold reduction compared to the scrambled control (kd cont.). (C) SAHH and DNMT1 transcription is efficiently reduced after siRNA mediated knock down in NT2 cells. Expression was quantified by qRT-PCR analysis in the knockdown experiment used for the COBRA analysis shown in figure 4D. Y-axis values indicate fold reduction compared to the scrambled control (kd cont.). (D) Western blot showing the effect of RA-treatment on the stability of EZH2 in NT2 cells during one month of treatment. Reduced protein levels could be observed after 3 weeks. β-actin was used as loading control. (5.08 MB TIF) [file pone.0010726.s003.tif]

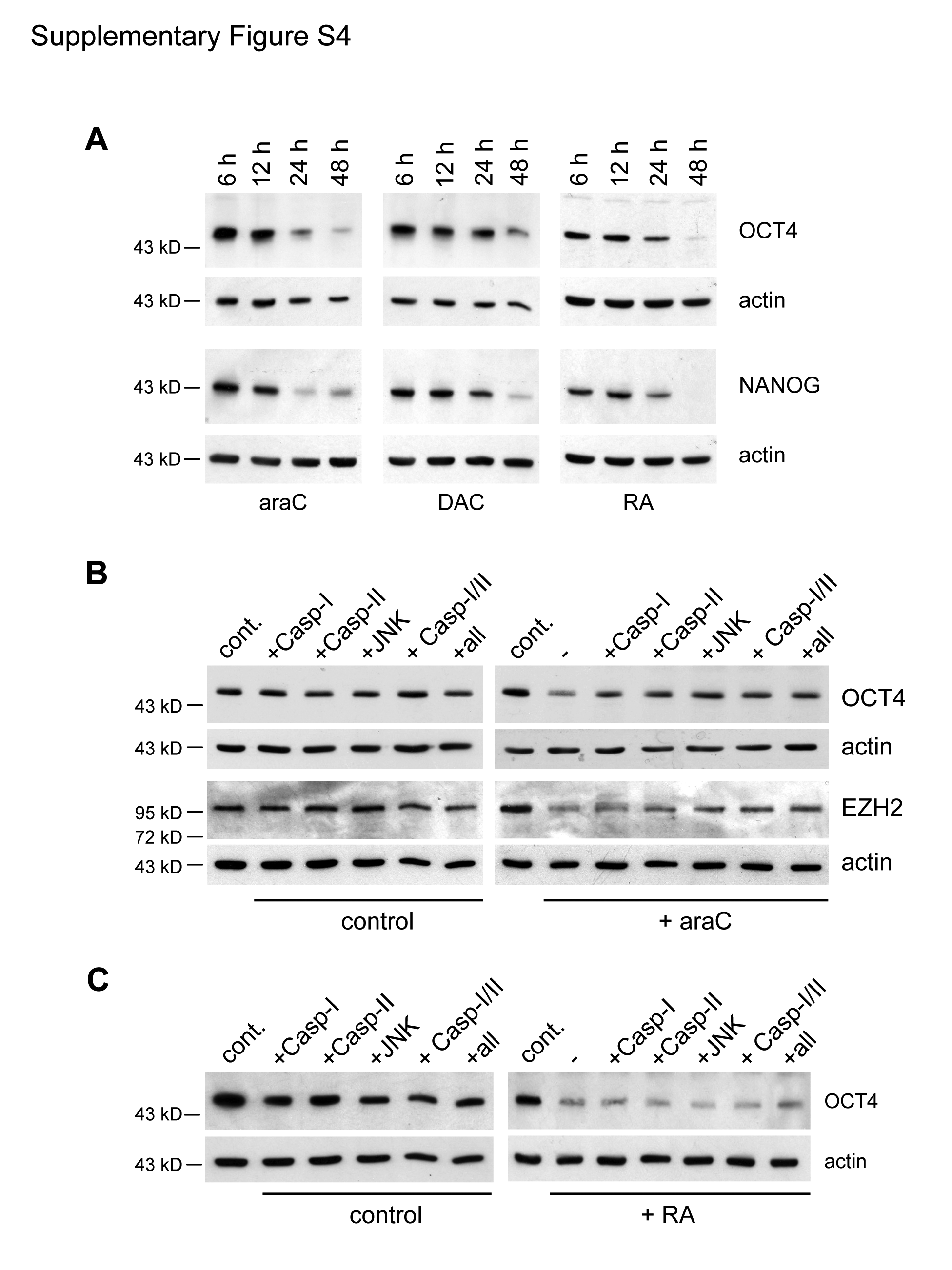

Supplement: Figure S4 — Differentiation-inducing drugs deplete NANOG and OCT4 by caspase-dependent degradation in NT2 cells. (A) Western blots showing levels of NANOG and OCT4 proteins in cells treated for 6, 12, 24 and 48 hours with araC, DAC and RA. β-actin was used as a loading control. The blot showing retinoic acid induced changes (on the right) was reprobed after NANOG-staining with OCT4 antibodies. Thus the β-actin control is the same for this experiment. DAC- and araC-treated OCT4 and NANOG blots are from two different experiments. (B) Western blots showing that drug-induced degradation of OCT4 and EZH2 in NT2 cells after 36 hours (OCT4) and 72 hours (EZH2) of treatment with araC is reduced (the signal is restored) by caspase or JNK inhibitors. β-actin served as loading control. (C) Western blots showing that RA-induced reduction of OCT4 48 hours of treatment with retinoic acid is not significantly reduced by caspase inhibitors I and II (Casp-I, Casp-II), JNK inhibitor 1 (JNK), a combination of both Caspase inhibitors (Casp-I/II) and a mixture of all three inhibitors (all). β-actin was used as loading control. (5.11 MB TIF) [file pone.0010726.s004.tif]

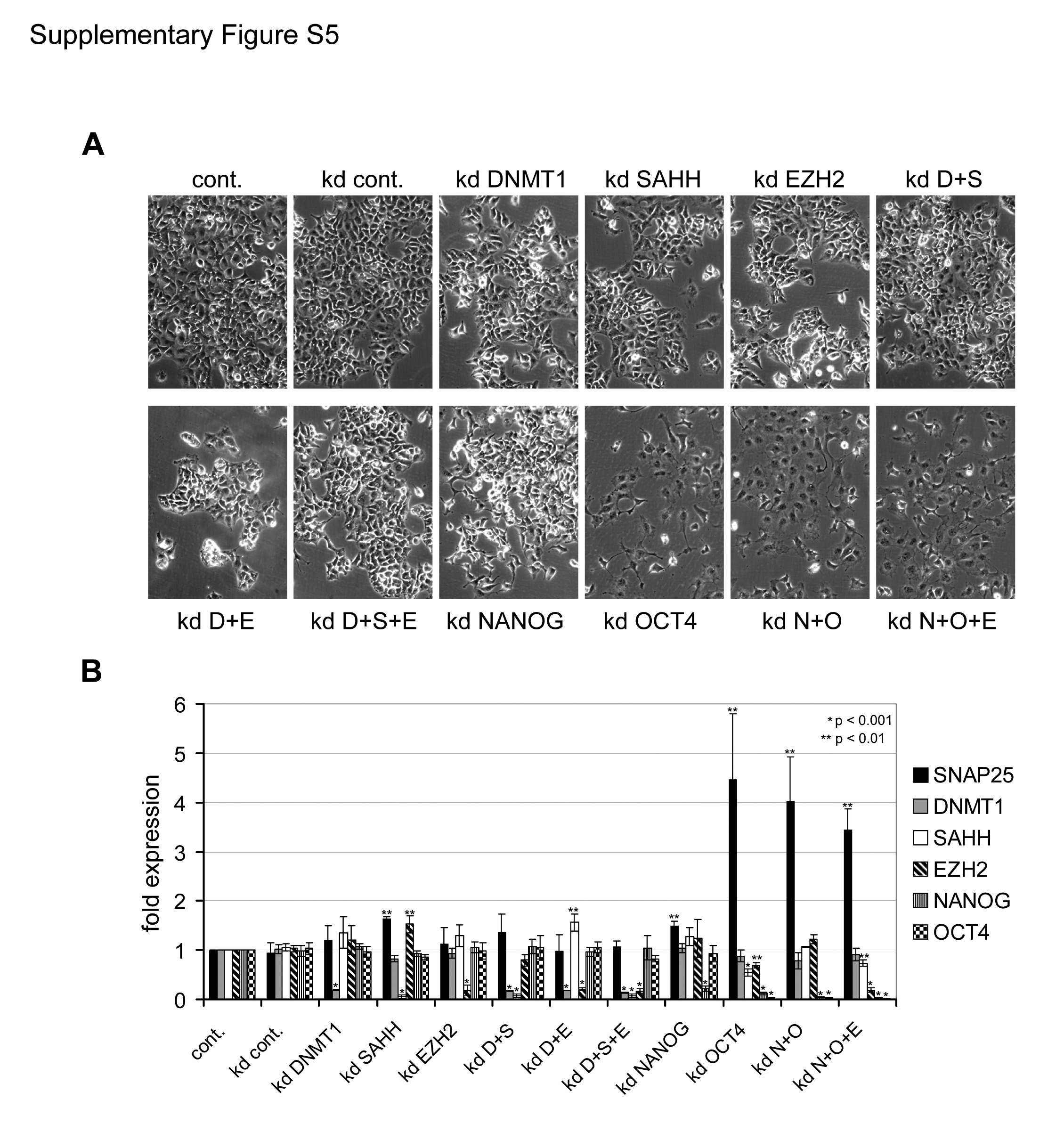

Supplement: Figure S5 — Depletion of stem cell factors induces differentiation of NT2 cells. (A) Microscopic images (10× magnification) of NT2 control cells (cont.) and NT2 transfected with a mix of scrambled siRNAs (kd cont.), and siRNAS specific for DNMT1 (D), SAHH (S), EZH2 (E), NANOG (N), OCT4 (O) and various combinations of these. After transfection the cells were grown for 72 hours. Knock down of OCT4 clearly induces neuronal phenotypes in NT2 cells. (B) Quantitative RT-PCR expression analysis of the differentiation marker SNAP25 and of DNMT1 (D), SAHH (S), EZH2 (E), NANOG (N) and OCT4 (O) after the knock down experiment described in (A). Diagrams show fold differences compared to control cells (cont.). The transcription of DNMT1, SAHH, EZH2, NANOG and OCT4 is efficiently reduced after the respective siRNA mediated knock down. Expression of the differentiation marker SNAP25 is weakly induced by NANOG knock down, but significantly upregulated upon depletion of OCT4, which is in line with the morphological observations. A combined knock down of NANOG and OCT4, or NANOG, OCT4 and EZH2 does not enhance the effect. qRT-PCR measurements are from two independent experiments and were internally normalised to the corresponding lamin-b and β-actin expression levels. P-values (Student's t-test) for the expression differences between untreated cells (cont.) and transfected cells for highly significant cases (p≤0.01) are indicated. Asterisks indicate expression values that are significantly different from controls. (5.49 MB TIF) [file pone.0010726.s005.tif]

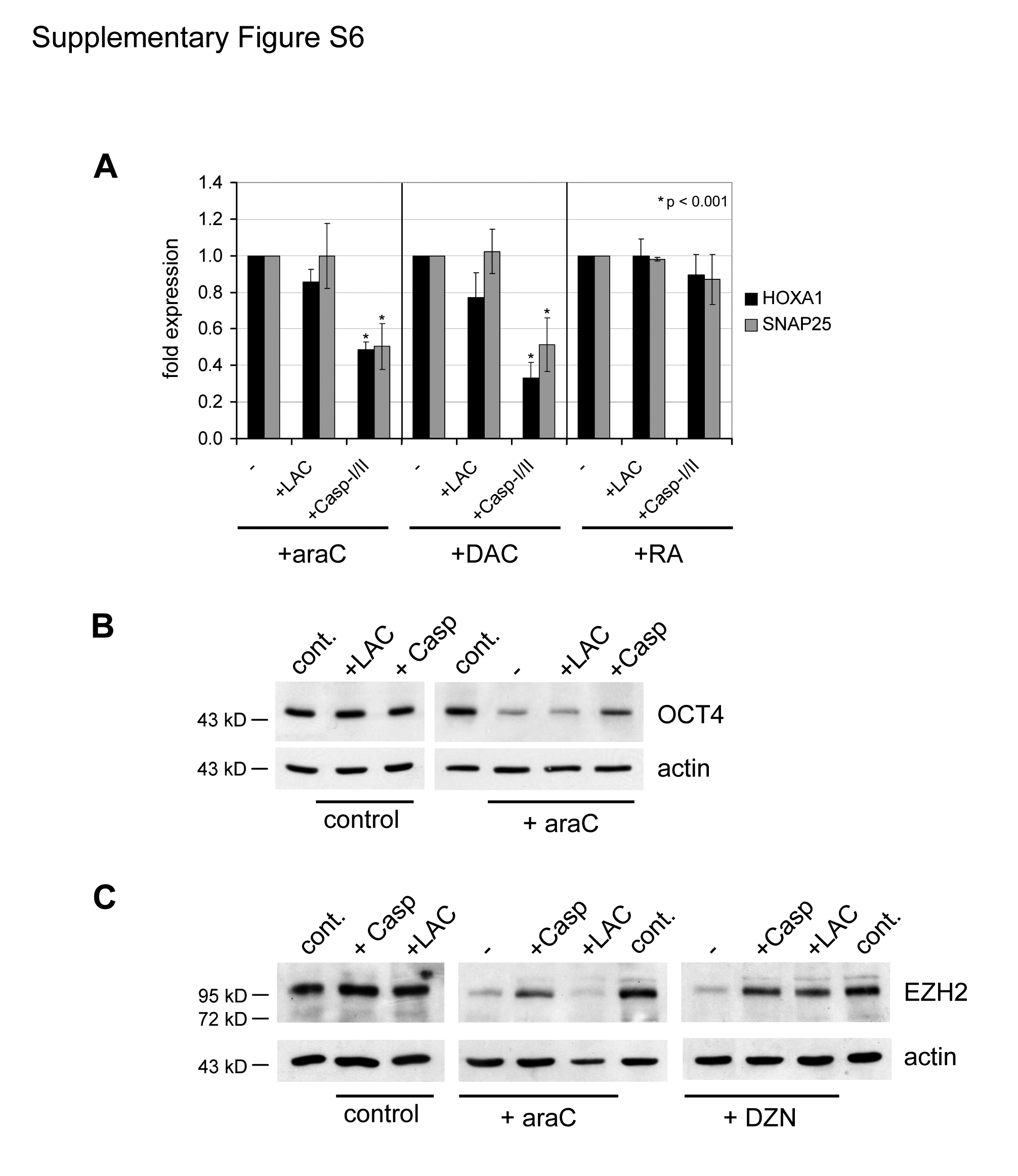

Supplement: Figure S6 — The effects of araC and DAC can not be rescued by the proteasome inhibitor Lactacystin. (A) Quantitative RT-PCR expression analysis of the differentiation markers SNAP25 and HOXA1 in NT2 cells after 3 days of treatment with araC, DAC or RA alone or in combination with the proteasome inhibitor Lactacystin (LAC) or a combination of two caspase inhibitors (Casp-I/II). Diagrams show fold differences compared to araC-, DAC- and RA-treatment alone. Co-treatment with caspase inhibitors leads to reduced gene expression after araC- and DAC-treatment, whereas Lactacystin had no significant effect. All qRT-PCR measurements were repeated at least three times and internally normalised to the corresponding lamin-b and β-actin expression levels. P-values (Student's t-test) for the expression differences between only drug-treated and co-treated cells for highly significant cases (p≤0.01) are indicated. Asterisks indicate expression values that are significantly different from controls. (B) Western blots showing that drug-induced degradation of OCT4 in NT2 cells after 36 hours of treatment with araC is reduced (the signal is restored) by the mix of caspase inhibitors (Casp), but not by treatment with Lactacystin (LAC, middle). β-actin served as loading control. (C) Western blots showing that drug-induced degradation of EZH2 in NT2 cells after 72 hours of treatment with araC is reduced (the signal is restored) by the mix of caspase inhibitors (Casp), but not by treatment with Lactacystin (LAC, middle). In contrast, DZNep-induced degradation of EZH2 is also inhibited by Lactacystin (blot on the right). β-actin served as loading control. (4.70 MB TIF) [file pone.0010726.s006.tif]

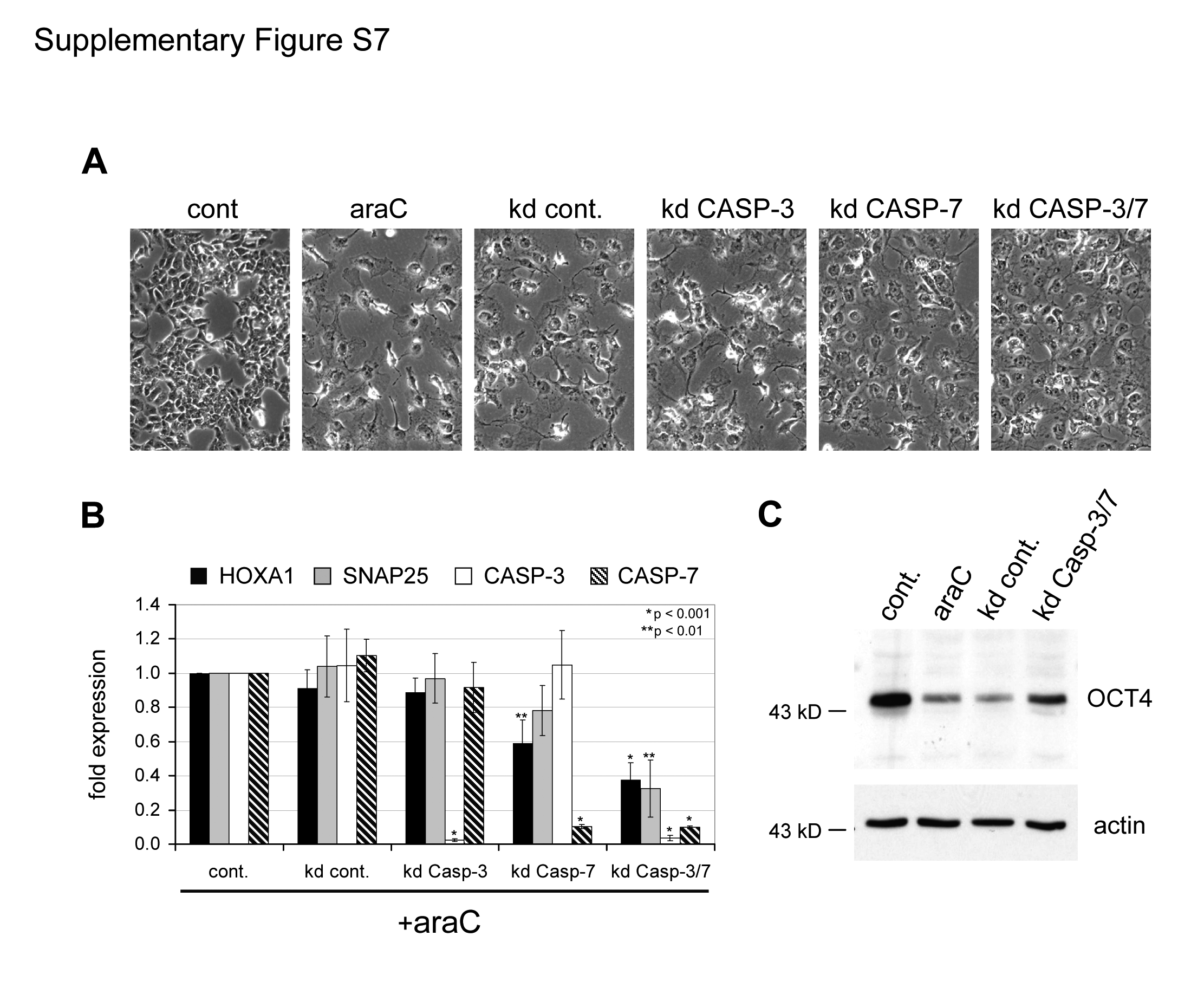

Supplement: Figure S7 — The effect of differentiation-inducing drugs in NT2 cells can be rescued by depletion of CASPASE-3 and -7. (A) Microscopic images (10× magnification) of NT2 control cells (cont.) and NT2 cells treated for 54 h with 10 µM araC (araC). The remaining images show cells that were treated for 6 hours with 10 µM araC, then were transfected with a mix of scrambled siRNAs (kd cont.), CASPASE-3 specific siRNAs (kd CASP-3), CASPASE-7 specific siRNAs (kd CASP-7) and a mix of both (kd CASP-3/7). After transfection the cells were grown for further 48 hours (with araC present). The araC-induced neuron-like morphology can be reverted to some degree by the double knock down. (B) Quantitative RT-PCR expression analysis of the differentiation markers SNAP25, HOXA1 and of CASPASE-3 and CASPASE-7 after the treatment described in (A). Diagrams show fold differences compared to araC-treatment alone. CASPASE-3 and CASPASE-7 transcription is efficiently reduced after the respective siRNA mediated knock down. Combined knock down of both caspases leads to a significantly reduced araC-induced expression of SNAP25, HOXA1. qRT-PCR measurements are from two independent experiments and were internally normalised to the corresponding lamin-b and β-actin expression levels. P-values (Student's t-test) for the expression differences between only araC-treated (cont.) and transfected cells for highly significant cases (p≤0.01) are indicated. Asterisks indicate expression values that are significantly different from controls. (C) Western blots showing that araC-induced degradation of OCT4 (under the conditions described in A) is reduced (the signal is restored) by combined depletion of CASPASE-3 and CASPASE-7. β-actin served as loading control. (4.08 MB TIF) [file pone.0010726.s007.tif]

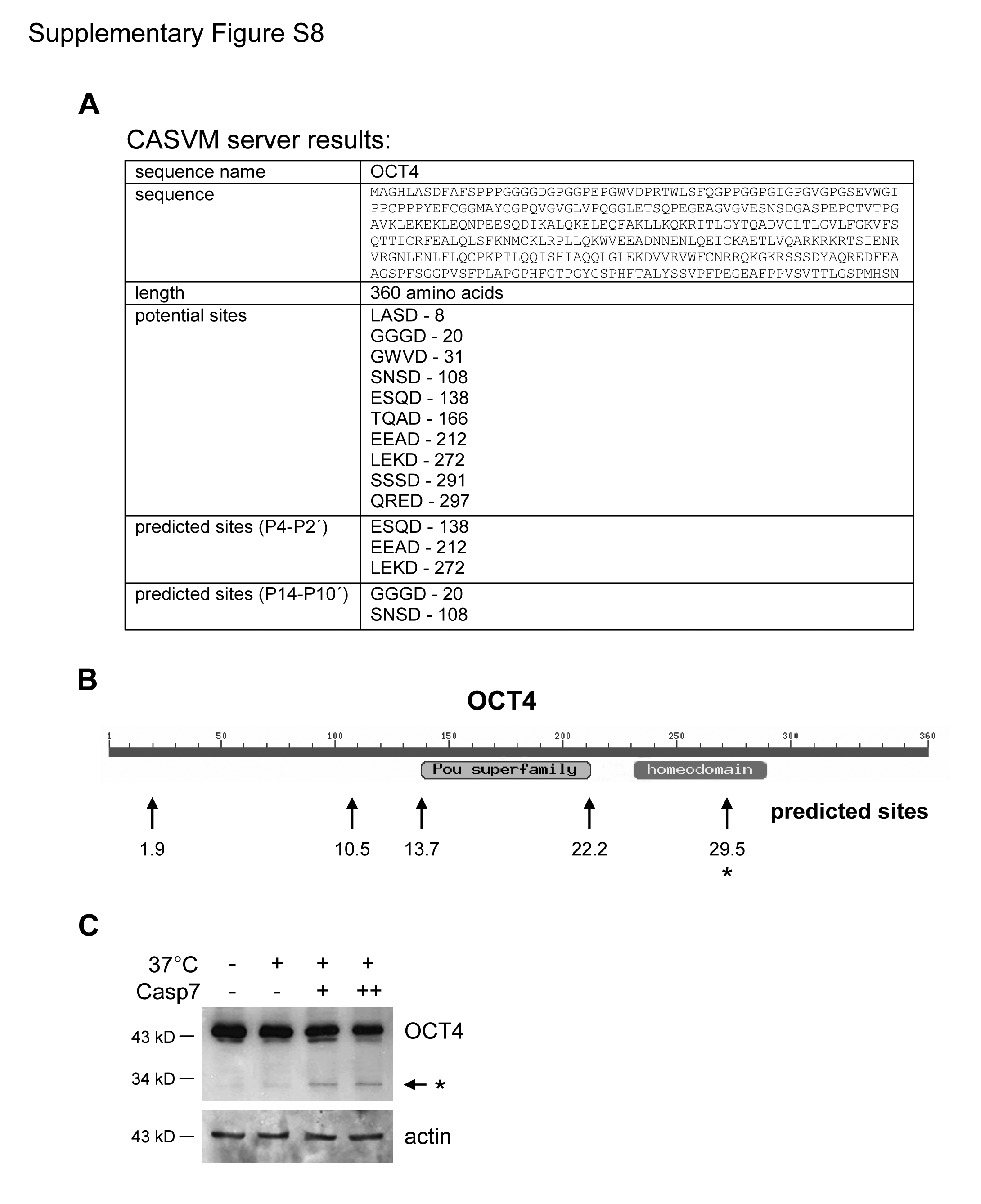

Supplement: Figure S8 — Potential caspase sites in the OCT4 protein. (A) Predicted caspase substrate cleavage sites by the CASVM (server for SVM Prediction of Caspase Substrates Cleavage Sites - http://casbase.org/casvm/index.html) using the P4-P2′and P14-P10′-trained classifiers are listed. The four amino acid core sequence (P4-P1) and the position of the P1-site along the 360 amino acid sequence of OCT4 are shown for each potential or predicted site. (B) The positions of the 5 predicted cutting sites are indicated below a sketch of the OCT4 protein. The POU-domain and the homeodomain are highlighted. The calculated molecular weight in kD for potentially originating N-terminal fragments after caspase digest are indicated below the arrows. The potential site used by CASPASE-7 is marked with an asterisk (C) Western blots showing the levels of OCT4 in nuclear extract from NT2 cells without treatment (cont.), in nuclear extract incubated at 37°C or treated with 1 (+) or 2 (++) units of active recombinant human Caspase-7 at 37°C. An antibody specific for an N-terminal peptide of OCT4 was used. β-actin was used as loading control. The blot is identical to the one shown in Fig. 7C but was exposed longer. A digestion product of ca. 30 kD becomes visible, that is significantly increased upon CASPASE-7 digestion (arrow with asterisk). This would correlate with a cut at the predicted site within the homeodomain of OCT4 (asterisk in B). (5.86 MB TIF) [file pone.0010726.s008.tif]
