# Supplementary material for: Nucleoside Drugs Induce Cellular Differentiation by Caspase-Dependent Degradation of Stem Cell Factors
Source: PLoS One. 2010 May 19;5(5):e10726. doi: 10.1371/journal.pone.0010726 (PMC2873290; doi:10.1371/journal.pone.0010726)
Supplement: Table S1 — RT-primer pairs used in this study. (0.04 MB DOC) [file pone.0010726.s009.doc]

RT-Primer pairs (5´-3´):

| HOXA1 | for  rev | GCCGTACTCTCCAACTTTC  CTCGCCTCAATACATTCACC |
| --- | --- | --- |
| HOXA2 | for  rev | TGCAGCATCTGAATTACTAAAAACA  CCAAATAAAAGAAGGCAAAACC |
| HOXA3 | for  rev | TGCTTTGTGTTTTGTCGAGACTC  CAACCCTACCCCTGCCAAC |
| HOXA4 | for  rev | ATGAAGAAGATCCATGTCAGC  CAGACAAACAGAGCGTGTGG |
| HOXA5 | for  rev | TGAAGTGGAACTCCTTCTCCAGC  CGCAAGCTGCACATAAGTCATG |
| HOXA6 | for  rev | tgggctgcgtggaattgatgagc  gatgcagcgcatgaactcctgcg |
| HOXA7 | for  rev | TCATTCCTCCTCGTCTTCC  GCCAATTTCCGCATCTACC |
| HOXA9 | for  rev | CACTCGTCTTTTGCTCGGTC  CAGCCAACTGGCTTCATGC |
| HOXA10 | for  rev | GACGCTGCGGCTAATCTCTAGG  AGCAAAGCCTCGCCGGAGAAG |
| HOXA11 | for  rev | gacaaggccggcggctcc  tgtatgaagccccccaccc |
| HOXA13 | for  rev | CGTCGTGGCTGATATCCG  GGGAGAAAGAAGCGCGTG |
| NEFL | for  rev | CTGCCAAGGCTGAGGAAGC  GTTCAATCTTTCTTCTTAGCTG |
| SNAP25 | for  rev | GCCAGATCGACAGGATCATG  CAACCTTTGGAAAGAAAGAGAC |
| NANOG | for  rev | CCTCAGCCTCCAGCAGATG  GGTCTGGTTGCTCCACATTG |
| OCT4 | for  rev | CTGACAACAATGAAAATCTTCAG  GTTACAGAACCACACTCGGAC |
| EZH2 | for  rev | GCTCAAGAGGTTCAGACGAG  CCTGATCTAAAACTTCATCTCC |
| CASPASE-3 | for  rev | GATCGTTGTAGAAGTCTAACTG  GAGCCATCCTTTGAATTTCGC |
| CASPASE-7 | for  rev | ACATGAATTTTGAAAAGCTGGG  CAGGCGGCATTTGTATGGTC |
| CASPASE-9 | for  rev | TTGTGTCCTACTCTACTTTCC  AAATCCCTTTCACCGAAACAG |
| p53 | for  rev | GCCAAGACCTGCCCTGTG  GATGGTGGTACAGTCAGAGC |
| JNKI | for  rev | GAGGTCATCCTTGGCATGG  ACAAATCCCTTGCCTGACTG |
| JNKII | for  rev | AGGTTATTCACATGGAGCTG  CAAAGTCAAGGATCTTCAGG |
| LAMINB1 | for  rev | CTGGAAATGTTTGCATCGAAGA  GCCTCCCATTGGTTGATCC |
| B-ACTINE | for  rev | GATCAAGATCATTGCTCCTCCTG  CTAGAAGCATTTGCGGTGGAC |
