# Supplementary material for: Nucleoside Drugs Induce Cellular Differentiation by Caspase-Dependent Degradation of Stem Cell Factors
Source: PLoS One. 2010 May 19;5(5):e10726. doi: 10.1371/journal.pone.0010726 (PMC2873290; doi:10.1371/journal.pone.0010726)
Supplement: Table S2 — COBRA primer pairs used in this study. (0.03 MB DOC) [file pone.0010726.s010.doc]

COBRA primer pairs (5´-3´):

| HOXA1 | for  rev | TAAGAATTTAGAGGGTGAAGGTTG  TCAACTATCCAAAACTTAAAAAAAA |
| --- | --- | --- |
| HOXA5-6 | for  rev | AGTTGAGAGGTAAGTGGAGTTTTTT  AAAAAAAATCTAAAAACTAAAACCC |
| HOXA6 | for  rev | GGGGTTTTTTTGTTTGTTATTGTT  CCTTCTTAAACCAACTACCCCTCTACC |
| HOXA7 | for  rev | GGTGAGAGAAGATTTGGGTA  CACCCCCAAATTTACACCAA |
| HOXA9 | for  rev | ATTATTGTTTAATTTTATGTGAGGG  AAAAACTAACCCAAAATCCC |
